# Mechanochemical and machine-intelligent design of programmable materials: From molecular interactions to macroscale functionality

**DOI:** 10.1016/j.mtbio.2026.103188

**Published:** 2026-05-08

**Authors:** Mitra Najafloo, Leila Naji, Christoph Eberl

**Affiliations:** a Department of Chemistry, AmirKabir University of Technology (Polytechnic), Tehran, Iran; b Cluster of Excellence livMatS @ FIT – Freiburg Center for Interactive Materials and Bioinspired Technologies, University of Freiburg, Freiburg, 79110, Germany

**Keywords:** Programmable materials, Mechanochemistry, Stimuli-responsive polymers, Smart materials, Machine intelligence

## Abstract

Programmable materials are an emerging class of matter capable of dynamically altering their properties, structure, or function in response to external stimuli. While most research has treated chemical and mechanical responsiveness separately, integrating these domains through mechanochemical design opens new avenues for intelligent, adaptive systems. This review explores how chemical reactivity and molecular interactions can be harnessed alongside mechanical deformation to create materials with controllable behavior across multiple scales. Key topics include force-activated molecular units (mechanophores), stress-guided chemical patterning, and materials whose structure-function relationships evolve under load. We highlight the role of machine intelligence in accelerating the discovery and optimization of programmable metamaterials, emphasizing inverse design, data-driven property prediction, and autonomous adaptation. Applications in soft robotics, shape-memory systems, self-healing materials, and smart coatings are discussed, focusing on chemomechanical feedback loops enhanced by computational tools. Multiscale modeling approaches that integrate chemical kinetics, mechanical stress analysis, and AI-guided generative design are also reviewed. By bridging polymer science, molecular chemistry, mechanical engineering, and artificial intelligence, this framework enables the design of materials that are not only responsive but predictive and self-evolving. Current challenges including scalability, reversibility, and durability are considered, alongside future directions toward biologically inspired, resilient material systems.

## Highlights

• Introduces a unified mechanochemical design framework integrating chemical reactivity with mechanical deformation.
• Reviews advances in force-activated molecular units (mechanophores) and stress-guided chemical patterning.
• Explores evolving structure–function relationships in load-adaptive programmable materials.
• Examines AI-driven inverse design, multiscale modeling, and data-enabled optimization of metamaterials.
• Discusses applications in soft robotics, shape-memory systems, self-healing materials, and smart coatings.
• Identifies key challenges including scalability, reversibility, durability, and autonomous adaptation.

## Graphical abstract

**Figure:**
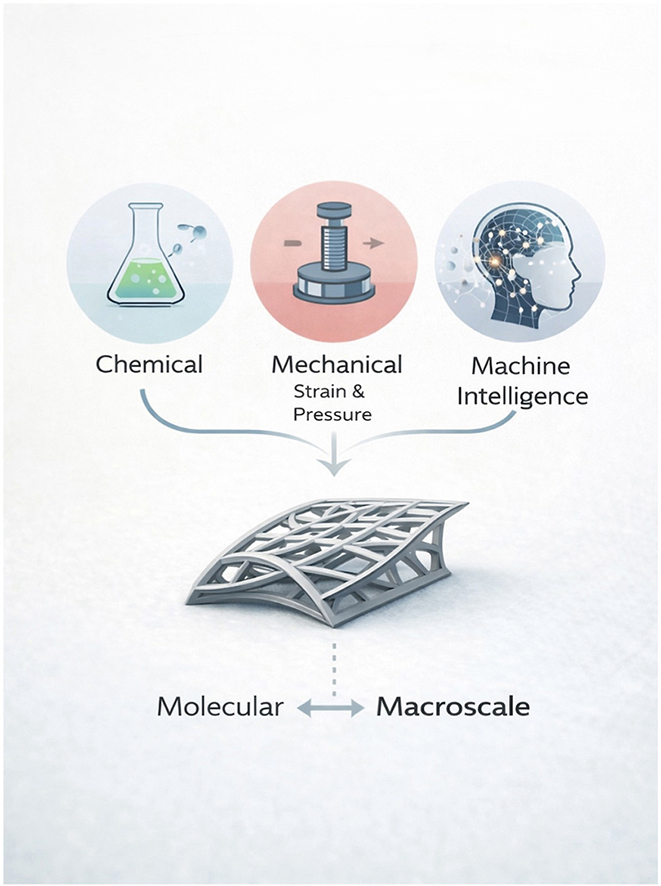

## 1 Introduction

The quest for materials that can adapt, respond, and evolve in a controlled manner under external stimuli has led to the emergence of programmable materials a class of systems whose internal structure or macroscopic behavior can be modified based on user-defined instructions. Unlike conventional passive materials, programmable materials exhibit dynamic properties such as shape-shifting, stiffness modulation, self-healing, or function switching [1,2]. Their programmability often depends on how they interact with thermal, electrical, magnetic, optical, chemical, or mechanical signals, giving rise to vast opportunities for intelligent systems in Refs. [3,4] (see Scheme 1).

**Schematic 1:**
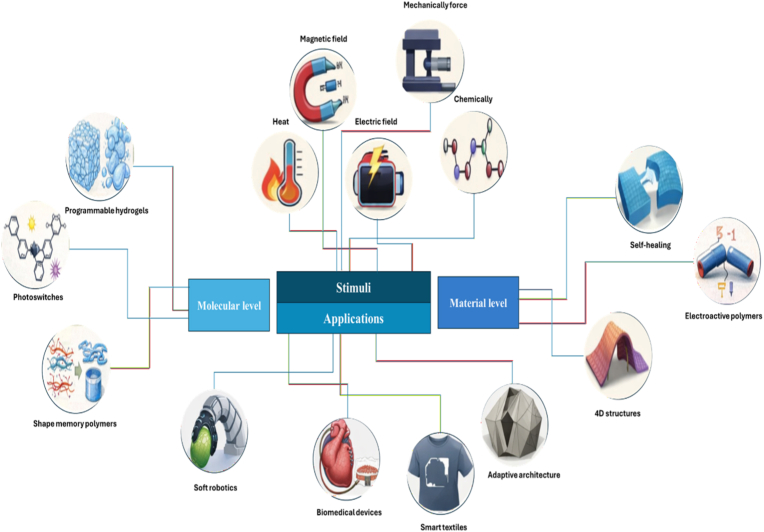
Overview of programmable materials and their stimuli.

Traditionally, the development of smart or responsive materials has followed two main directions: chemically-driven systems (e.g., Ref. [5], redox-switchable polymers) and mechanically-activated systems (e.g., shape-memory alloys, strain-tunable metamaterials) [[6], [7], [8]]. However, emerging research reveals that the intersection of mechanics and chemistry an interdisciplinary field often termed mechanochemistry can unlock new design paradigms for advanced, multifunctional materials [9,10]. Mechanochemistry explores how mechanical forces influence chemical reactions and vice versa, enabling feedback systems where materials can both sense and adapt to their mechanical environment [11].

The integration of mechanochemical concepts into material design is not only scientifically exciting but also functionally powerful. It allows the programming of responses at multiple scales, from molecular conformational changes to macroscopic structural reconfigurations [12]. For example, embedding force-responsive chemical bonds (mechanophores) in polymer backbones enables stress-induced color changes, bond cleavage, or network remodeling all of which are reversible and programmable with external stimuli [13,14].

Historically, mechanochemistry was largely limited to niche chemical transformations or destructive material failure studies. Yet in the past two decades, mechanochemistry has matured into a design tool for developing constructive, adaptive, and reversible material functionalities. The invention of self-healing polymers, soft robotics with mechanical memory, and drug carriers responsive to mechanical environments exemplifies this transformation [[15], [16], [17]]. Concurrently, computational tools have enabled the design of materials with tunable mechanochemical properties by simulating stress distributions, reaction kinetics, and structural evolution under applied forces.

Interdisciplinary research at the interface of polymer science, solid mechanics, molecular chemistry, and materials engineering has led to breakthroughs in creating hierarchical materials that can “learn” or evolve behavior through external programming [18,19]. In particular, the rise of 4D printing, where time-dependent behavior is encoded into printed structures, relies heavily on chemomechanical principles [[20], [21], [22]]. These materials can undergo programmed shape changes, self-assemble, or repair themselves upon specific mechanical or environmental triggers.

Beyond experimental and mechanochemical strategies, machine intelligence is emerging as a powerful accelerator in the design of programmable materials. The integration of artificial intelligence (AI) and data-driven modeling enables researchers to move beyond trial-and-error synthesis toward predictive, inverse design strategies [23]. By learning from large datasets of structural motifs, property measurements, and simulation outputs, machine learning (ML) algorithms can identify hidden correlations between chemical composition, microstructural geometry, and macroscopic performance [[24], [25], [26]]. In metamaterials, AI-driven generative design has already been used to optimize architectures for extreme stiffness-to-weight ratios, unusual thermal expansion, or tailored acoustic and photonic responses. For programmable systems, this convergence of mechanochemistry with machine intelligence creates a pathway toward autonomous materials that can sense, adapt, and even reconfigure themselves with minimal human intervention [27,28]. Neural networks and evolutionary algorithms can propose new mechanophore chemistries or polymer backbones optimized for stress-induced responses, while reinforcement learning schemes can suggest adaptive actuation patterns in soft robotic platforms. Furthermore, the concept of digital twins virtual replicas of materials that continuously update with experimental or in situ performance data offers a blueprint for real-time optimization of programmable matter [29].

Programmable materials have found increasing applications in various disciplines. In soft robotics, they enable actuators and grippers that adapt to diverse environments [[30], [31], [32]]. In biomedicine, mechanochemically responsive drug delivery systems can release therapeutic agents in response to body movements or tissue mechanics [[33], [34], [35]]. In structural engineering, adaptive facades and deployable systems can be created that react to mechanical loading or environmental changes [36]. Even in wearable electronics, chemomechanically tunable substrates are enabling next-generation flexible sensors and displays [37,38]. Despite the exciting progress, several challenges remain in the practical deployment of mechanochemically programmable materials. These include scalability of synthesis, long-term stability, reversibility of responses, and predictability of behavior under complex loading conditions. Addressing these challenges requires a unifying framework that blends experimental insights with multiscale modeling, enabling a deeper understanding of how molecular interactions govern large-scale responses. This review aims to provide a comprehensive and interdisciplinary overview of the mechanochemical design of programmable materials, from molecular interactions to macroscopic applications (Schematic 1). We begin by laying out the fundamental principles of programmability, followed by recent advances in mechanochemical coupling, modeling strategies, and real-world case studies. Our goal is to highlight how mechanics and chemistry, when co-designed, offer unprecedented opportunities for building materials that are not only smart but also predictive, autonomous, and adaptable. In addition, we emphasize the emerging role of machine intelligence in accelerating metamaterials design, which provides new opportunities for inverse design and adaptive material functionalities.

## 2 Fundamentals of programmable materials

Before diving into the complexities of mechanochemical design, it's important to first understand what makes a material “programmable” in the first place. This section lays the foundation by unpacking how materials can be engineered to respond predictably to different external cues like heat, light, pressure, or chemical changes and what determines the nature and duration of that response. Not all programmable materials behave the same: some are designed to react once and retain that change permanently, while others can cycle between states, adapting repeatedly over time. We also explore the difference between static systems, which follow fixed instructions, and dynamic ones that evolve, reconfigure, or even “learn” from their environment. These distinctions aren't just academic they directly influence how materials are selected, designed, and applied in everything from soft robots to responsive biomedical devices.

### 2.1 Types of stimuli-responsive programmable materials

Programmable materials derive their functional behavior from their ability to respond in a predictable and often reversible manner to external stimuli. These stimuli can originate from various physical, chemical, or environmental sources and cause a controlled transformation in the material's properties such as shape, phase, conductivity, or optical characteristics [[39], [40], [41]]. The specific mechanism through which a material responds depends on its molecular structure, bonding interactions, and overall architecture, which determine how the material perceives and processes external signals.

**Fig. 1:**
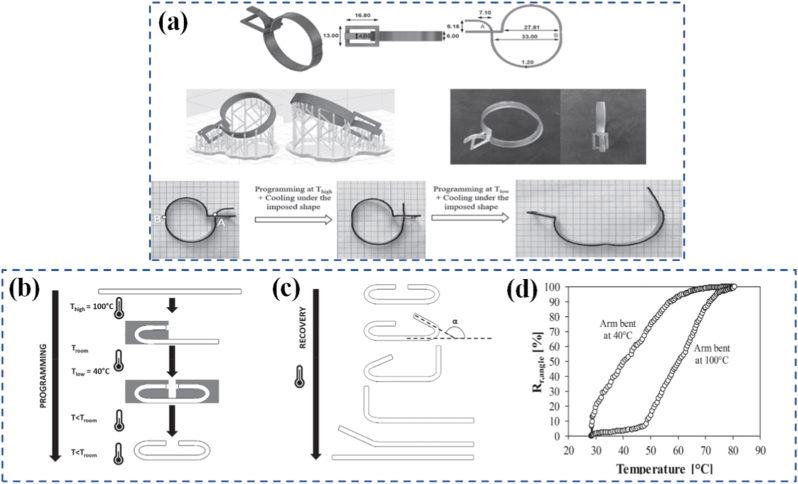
(a) Programmable self-locking clamp, (b) Outline of the programming, (c) The recovery stage (the angle α used to evaluate the shape recovery of the arm bent at *T*_low_, and (d) Recovery along a heating ramp of a bar-shaped specimen in its as-printed state, with one arm folded at *T*_high_ = 100 °C and at the other at *T*_low_ = 40 °C [47].

Thermally responsive materials are among the most widely studied due to the ubiquity and controllability of heat as a stimulus. These materials typically rely on temperature-induced phase transitions or glass transition behaviors [42]. For example, shape-memory polymers (SMPs) can be programmed into a temporary shape and later recover their original configuration upon heating past a specific temperature [43]. Some materials demonstrate advanced shape memory behaviors, such as polymers capable of triple or quadruple shape recovery by incorporating two or more distinctly separated transition temperatures. Others display a temperature-memory effect (TME), in which the recovery occurs over a broad range of transition temperatures, as well as hydrogels that can achieve triple shape memory [[44], [45], [46]]. In the case of TME, the material's permanent shape is restored from its programmed temporary forms at specific temperatures. These recovery temperatures match those applied during the material's programming stage and are often termed “deformation temperatures”. For instance, Inverardi et al. [47] showcased the applicability of the temperature-memory effect by creating a 4D-printed clamp with a self-locking function (Fig. 1(a–d)). In their approach, the clamp was sequentially programmed at two distinct deformation temperatures (*T*_high_ and *T*_low_) to establish two separate temporary configurations, after which its isothermal recovery behavior was evaluated by placing the device in a heated water bath. In another study, Guo et al. [48] developed liquid crystal elastomers capable of being reprogrammed via the shape memory effect, followed by reversible actuation of the reprogrammed shape triggered by both temperature changes and photothermal stimulation. Liu et al. [49] developed conductive fibers by incorporating a gradient of liquid metal fillers within a polymer matrix. These fibers exhibited shape programmability controlled by the thermal response of the liquid metals, alongside tunable electrical conductivity. When heated, they transitioned from an insulating to a conducting state, with the conductivity profile predetermined along the fiber's length. Both the shape memory behavior and the conductivity switching could be repeatedly achieved through thermal cycling. However, it should be noted that thermally driven methods may be unsuitable for systems containing volatile droplets, as evaporation or reduced measurement reliability can occur [50]. Similarly, thermochromic materials change color in response to temperature, which can be exploited in smart labels or thermal sensors [[51], [52], [53]]. The nature of the thermal response whether it is elastic, viscous, or molecular can be precisely tailored using polymer blends, nanocomposite design, or molecular engineering. Punpongsanon et al. [54] fabricated objects with a voxel-based surface design, where each voxel was assigned a distinct photochromic color. To achieve the target visual pattern, voxels with undesired colors were deactivated using visible light, while those contributing to the design were activated with ultraviolet light. Addressing the drawbacks of this method such as limited resolution, a restricted color palette, and the dependence on multi-material 3D printing Jin et al. [55] ntroduced an alternative strategy. They incorporated photochromic dyes into a single homogeneous solution, enabling the production of reprogrammable, multi-colored textures from one material. These dyes could switch between a transparent state and a colored state under illumination with specific wavelengths.

**Fig. 2:**
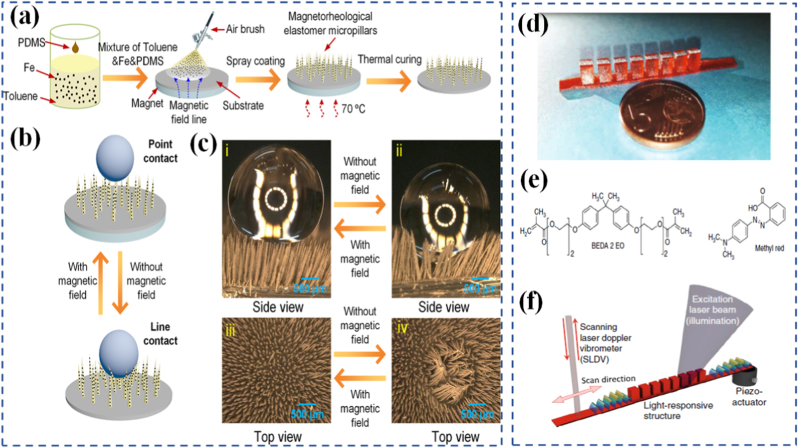
(a) Schematic illustration of the fabrication procedure of the magnetically responsive superhydrophobic surface, (b) Scheme for reversible switching of the wettability and adhesion properties of the magnetically responsive superhydrophobic surface by on/off switching of magnetic field, (c) Optical microscope images showing the stiffness tunability of the MREMPs under an external magnet field, which can transform the micropillars from the collapsed morphology (water-adhesive state) to the fully upright position (water-repellent state) [62], (d) Picture of the LRMM patterned with periodic square pillars, (e) Molecular structures of the azopolymer, and (f) Sketch of the experimental configuration in which a piezoactuator excites elastic waves in the slab, a 405 nm laser beam is used to induce a spatially selective photo-softening effect depending on the illuminationarea and a 633 nm laser vibrometer scans the whole structure while detecting point-by-point the out-of plane oscillation amplitude [63].

Magnetic-responsive materials integrate magnetic particles or domains that respond to applied magnetic fields, which can actuate motion, change stiffness, or align internal structures [56]. These are highly advantageous in remote-control applications such as micro-robotics or drug delivery systems, where contactless manipulation is essential. Depending on the composition and domain structure, the response may be instantaneous and reversible or require thermal or chemical locking mechanisms to hold a specific shape [57,58]. The ability to spatially and temporally control magnetic fields also enables fine-tuned actuation across multiple degrees of freedom. Qi et al. [59] produced magnetic filaments composed of polylactic acid (PLA) and carbonyl iron particles using extrusion-based 3D printing, later embedding these components into silicone rubber. The use of 3D printing allowed precise control over the geometry, spatial arrangement, and alignment of the magnetic elements, enabling the creation of anisotropic magnetization patterns that facilitated programmable shape transformations. Furthermore, the applied stimulus could modulate both the stiffness and electromagnetic characteristics of the material across several orders of magnitude [60,61]. In a related study, Yang et al. [62] examined how magnetic fields influence the wettability and adhesion of surfaces featuring magnetorheological elastomer micropillars. As illustrated in Fig. 2(a), these surfaces were fabricated to reversibly adjust their interfacial properties by toggling the magnetic field on and off. Optical microscopy images in Fig. 2(b, c) highlight how magnetic actuation can alter the stiffness of the micropillars, shifting their behavior from retaining water droplets to repelling them.

Optically responsive materials change their behavior under illumination and are especially useful when spatial and temporal control is desired without physical contact. Light can trigger reversible photoisomerization, crosslinking, or degradation of molecular bonds, leading to changes in mechanical, optical, or chemical properties [64,65]. Azobenzene-functionalized polymers, for instance, undergo reversible trans–cis isomerization when exposed to UV or visible light, enabling controlled shape deformation or property modulation. These light-driven materials are crucial in data storage, optoelectronics, and reconfigurable surfaces [66,67]. Gliozzi et al. [63] utilized a commercial digital light processing 3D printer to fabricate elastic metamaterials by curing a UV-sensitive resin doped with methyl red, an azo-based compound. These structures exhibited a controllable frequency band gap width. As shown in Fig. 2(d and e), the experimental arrangement involved a piezo-actuator to generate elastic waves within the slab, while a 405 nm laser provided localized photo-softening in regions determined by the illumination pattern. A scanning laser Doppler vibrometer operating at 633 nm was then employed to map the structure, recording the out-of-plane vibration amplitude at each point (Fig. 2(f)). The resulting spectral characteristics and dispersion curves were subsequently analyzed.

**Fig. 3:**
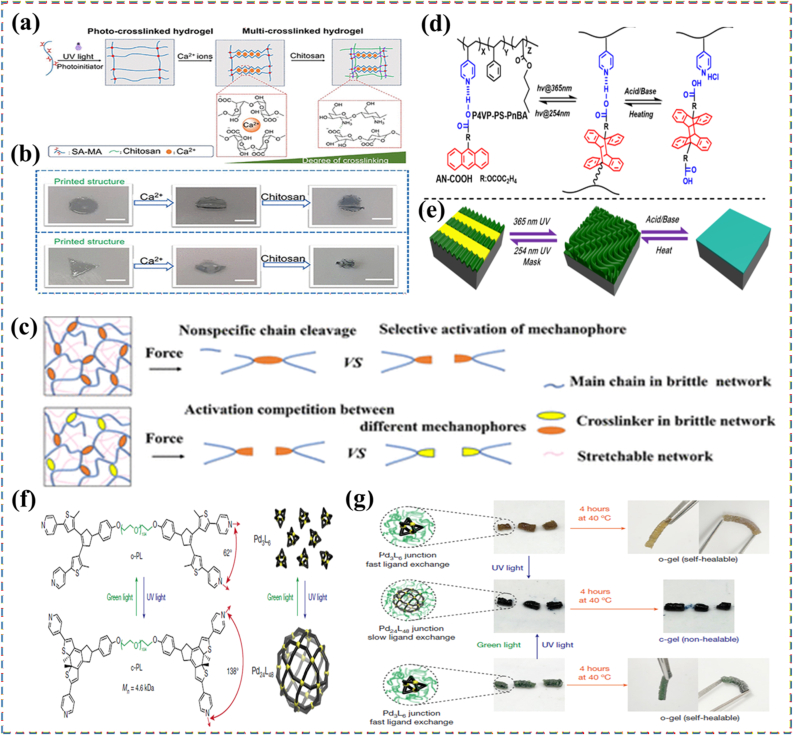
(a) Deformation mechanism of photocrosslinked methacrylated alginate, (b) Step-wise deformation of 3D-printed SA-MA5 hydrogels [72], (c) Illustration of the competition between the selective activation of the mechanophore in the cross-linker and nonspecific bond rupture in the main chain backbone [73],(d) Chemical structure and reaction of the top layer materials during the process of wrinkling pattern generation/Elimination, (e) Schematic illustration of selective generation/elimination of the wrinkling patterns and response to base/acid [73], (f) Chemical structure of the photoresponsive polymer ligand and a schematic of MOC interconversion, and (g) Photographs of self-healing experiments, which demonstrate that the o-gel undergoes self-healing whereas the c-gel does not [74].

Chemically and mechanically responsive materials often interact in deeply coupled ways. Chemically responsive systems react to changes in pH, ionic strength, oxidation state, or solvent environment, enabling swelling, contraction, or bond reorganization [[68], [69], [70]]. Hydrogels that expand in acidic or basic conditions are examples used in biosensors and actuators. Zhou et al. [71] eveloped programmable hydrogel structures using a dual shape-programming approach, combining polyacrylamide/gelatin with poly(acrylamide-co-methacrylic acid)/gelatin networks. In this method, supramolecular assembly served as the first programming stage to establish the hydrogels' initial anisotropy, while a shape memory process acted as the second stage to introduce temporary anisotropy. The resulting materials could react not only to temperature changes but also to pH variations, owing to the presence of carboxylic acid groups. Another example of a hydrogel with chemical responsiveness was created from methacrylated sodium alginate, designed for 3D printing and capable of reacting to both Ca^2+^ ions and chitosan [72]. This was achieved by grafting methacrylate groups onto sodium alginate chains, which were subsequently cross-linked at the graft sites. Additional cross-linking was possible through the introduction of Ca^2+^ or chitosan (Fig. 3(a)), enabled by strong interactions between alginate and these additives: Ca^2+^ formed ionic bonds with alginate's carboxylate groups, and chitosan's protonated amines could interact with the same sites. Immersing the gel in either Ca^2+^ or chitosan solutions increased its cross-link density and caused volumetric shrinkage, while sequential exposure to both stimuli produced a greater effect than either alone. The team also fabricated a bilayer hydrogel in which each layer had a distinct initial cross-link density due to variations in methacrylation degree. When placed in a Ca^2+^ solution, this bilayer bent as the less cross-linked layer contracted more significantly, producing a programmed shape change (Fig. 3(b)).

Mechanically responsive materials, on the other hand, use applied force or strain as the trigger. Mechanically responsive materials often rely on the activation of molecular mechanophores, which undergo specific chemical reactions when subjected to defined force thresholds. These mechanophores can cleave, rearrange, or form new bonds in response to stress, effectively converting mechanical input into chemical output. The type of reaction depends on the mechanophore's molecular structure and the force direction and magnitude. For instance, certain spiropyran-based mechanophores undergo reversible ring-opening under tensile stress, leading to colorimetric changes, while others can trigger bond cleavage to initiate polymer network rearrangements or self-healing processes. To aid understanding, schematic illustrations have been added (Fig. 3(c)), showing the force-induced bond activation pathways and subsequent macroscopic material responses. Incorporating these mechanistic details highlights how mechanical forces can be harnessed to drive precise chemical transformations and programmable material behavior [75]. A more quantitative understanding of mechanochemical coupling requires consideration of how external forces modify the reaction energy landscape along specific reaction coordinates. In mechanophore-containing systems, applied force can lower activation energy barriers and shift reaction pathways depending on both the magnitude and direction of the force relative to the molecular backbone. Factors such as force loading rate and spatial distribution of stress significantly influence reaction kinetics, where rapid loading may promote bond scission, while slower or distributed forces may favor reversible bond rearrangements. At the molecular level, efficient force transduction depends on polymer chain alignment and connectivity, as these determine how stress is transmitted to force-sensitive bonds. Increasing chain orientation or tailoring cross-linking density can enhance force focusing along specific pathways, thereby improving the selectivity and efficiency of mechanochemical activation. Bridging this molecular behavior to macroscale response requires hierarchical design strategies, where microstructural organization governs how local forces accumulate and trigger global material transformations. In advanced systems, the stress applied can activate molecular mechanophores, which cleave or rearrange under specific force thresholds [76,77]. These force-sensitive reactions may serve as damage indicators or initiate repair mechanisms, transforming stress from a failure mechanism into a functional input [78]. Zhang et al. [79] developed a molecular-scale, mechanically responsive biocatalytic platform embedded within hydrogels, enabling modulation of their macroscopic mechanical characteristics. When subjected to repeated tensile loading, these hydrogels could either increase or decrease their stiffness, while bilayer configurations demonstrated programmable shape transformations in response to mechanical forces. Recent advances have also highlighted a distinct class of stimuli-responsive systems that are activated by cellular contractile forces, bridging material science with mechanobiology. In these systems, living cells act as active mechanical agents that generate endogenous forces capable of driving material deformation and reconfiguration. For instance, recent studies on 4D bioprinted living constructs have demonstrated that cell contractile forces can induce spatiotemporal morphogenesis, enabling programmed shape transformations without external stimuli [80,81]. Similarly, cell-mediated morphogenetic tissue engineering using 4D printed degradable hydrogel scaffolds has shown that cellular traction forces can guide structural evolution and functional maturation of engineered tissues [82]. These approaches introduce a paradigm in which materials are not only responsive to external physical or chemical inputs but can also adapt dynamically to internal, biologically generated forces. Incorporating such cell-responsive mechanisms expands the scope of programmable materials toward bio-integrated and self-evolving systems, particularly in tissue engineering and regenerative medicine. It is important to highlight that some physical stimuli can be delivered remotely without direct material contact, whereas chemical stimuli generally require direct interaction to elicit a response. Achieving highly localized, small-scale, and time-dependent modifications within a material remains challenging [20]. Nonetheless, progress in material design at the micro- and nanoscale is making it feasible to induce controlled, localized volume variations [83]. Additionally, coupling chemical responses with physical triggers offers a pathway to overcome existing constraints for instance, photoacid compounds can alter local pH when illuminated with light. In parallel, advanced characterization methods are being developed to monitor ion movement within battery electrodes, potentially enabling precise spatiotemporal control over internal changes [84]. Future studies should aim to integrate such emerging strategies with material programmability to unlock new functional capabilities. A comparative summary of different stimuli, their mechanisms, advantages, limitations, and representative applications is provided in Table 1.

**Table 1:** Summary of stimuli-responsive programmable materials.

Stimulus Type	Mechanism	Advantages	Limitations	Representative Applications
Thermal	Phase transition, glass transition, shape memory effect	Easy to apply, well-understood, reversible	Slow response, energy consumption, not suitable for heat-sensitive systems	Shape-memory devices, soft actuators, smart textiles
Magnetic	Magnetic particle alignment, magneto-mechanical actuation	Remote control, fast response, precise spatial actuation	Requires magnetic field setup, material complexity	Soft robotics, targeted drug delivery, micro-actuators
Optical	Photoisomerization, photothermal effect, photocrosslinking	High spatial and temporal resolution, contactless	Limited penetration depth, potential photodegradation	Data storage, optoelectronics, reconfigurable surfaces
Chemical	pH change, ionic interaction, redox reactions	High sensitivity, suitable for biological environments	Slow diffusion, limited spatial control	Biosensors, drug delivery, smart hydrogels
Mechanical	Force-induced bond activation, mechanophores	Direct response to stress, damage sensing capability	Requires physical contact, difficult to localize precisely	Self-healing materials, stress sensors, adaptive structures
Biological (Cell-responsive)	Cell contractile forces, cell–matrix interactions	Autonomous, biologically driven, suitable for tissue integration	Complex control, variability in cellular behavior	Tissue engineering, 4D bioprinting, regenerative medicine

### 2.2 One-time vs. reversible programming

Programmable materials can be distinguished not only by how they respond to stimuli but also by how permanent or transient those responses are. This distinction is essential in determining the material's application domain, service lifetime, and operational flexibility. A foundational classification within this context is whether the programming is one-time (irreversible) or reversible, which directly impacts the material's recyclability, adaptability, and suitability for long-term or evolving use [85]. One-time programmable materials are designed to undergo a permanent transformation upon activation. This may involve chemical degradation, irreversible phase transition, or structural locking [86]. Such transformations are often used in transient electronics, biodegradable scaffolds, or deployable aerospace structures, where a one-off function is sufficient or even desired. For example, polymers that irreversibly depolymerize under UV light can be used for secure data storage or temporary implants that dissolve after their function is complete. In these cases, the material's response is not meant to be repeated but instead to deliver a precise, high-impact change with minimal external control [87].

Irreversible systems often rely on covalent bond breaking or chemical decomposition, which can be energetically favorable and stable under ambient conditions but lack reusability. While this limits their use in systems that require longevity or adaptive behavior, it makes them highly effective in mission-critical applications, such as mechanically triggered drug release, emergency self-deployment devices, or self-destructing sensors. Their reliability lies in the guarantee that once triggered, the change is definite and cannot be undone by environmental fluctuation [88,89]. At present, the functionalities and performance characteristics attainable through available chemical approaches remain somewhat constrained, particularly when aiming for properties such as biocompatibility, biodegradability, optical clarity, self-repair capability, and chemical stability. One promising direction would be the creation of hydrogel systems that can autonomously regulate their behavior in response to specific enzymes while also being programmed to react differently depending on enzyme concentration. Such adaptability would be especially valuable given that enzyme expression can vary significantly between different diseases and across the progression stages of the same condition. Current studies also reveal that polymer-based materials often undergo irreversible transformations after enzymatic action. Designing systems capable of reversing these changes could open the door to controlled, repeatable release of therapeutic molecules on demand [90,91].

In contrast, reversible programmable materials are capable of multiple cycles of transformation, often toggling between two or more stable or metastable states. These materials form the basis of smart sensors, reconfigurable electronics, and adaptive clothing, where repeated activation is essential. The reversibility can be thermally, chemically, optically, or mechanically controlled. Hydrogels that swell and shrink in response to repeated pH cycles, or light-switchable polymers that toggle their shape with alternating wavelengths, are representative examples [92,93]. The design of these materials often involves dynamic covalent bonds, supramolecular interactions, or physically induced phase changes, which offer the energy landscape required for cycling between states. Importantly, the choice between one-time and reversible programming is not simply a matter of performance, but of design philosophy [94]. It reflects trade-offs in material complexity, cost, response time, energy requirement, and resilience. The development of hybrid systems where certain responses are reversible while others are locked after activation is an emerging trend aimed at combining the best of both worlds, such as having a shape-shifting actuator that locks into place after deployment but can still provide real-time sensing or feedback [95]. Li et al. [73] investigated a dual-responsive anthracene-based supramolecular network that reacted to both light and HCl gas. The material's cross-linking relied on two cooperative interactions: anthracene photodimerization and hydrogen bonding between pyridine units on the monomers and carboxylic acid groups on the anthracene molecules (Fig. 3(d)). Designed for smart display surfaces, it could produce wrinkled surface patterns under 365 nm UV irradiation and erase them with 254 nm UV light (Fig. 3(e)). The reversible formation and cleavage of anthracene dimers under these wavelengths drove the patterning process. The wrinkles could also be removed by introducing HCl gas, which disrupted the hydrogen bonding network; heating the material expelled the HCl, restoring the original state. In another study, Gu et al. [74] developed a light-responsive supramolecular polymer network in which the cross-links were metal-organic cages (MOCs) containing organic polymer ligands that functioned as photoswitches. These ligands incorporated bis-pyridyl dithienylethene units, a type of diarylethene, capable of reversible ring-opening and ring-closing upon exposure to specific light wavelengths (Fig. 3(f and g)). This design allowed the network to reversibly toggle between two distinct topological configurations.

### 2.3 Static vs. dynamic programmability

Before distinguishing static and dynamic programmable materials, it is important to clarify their conceptual difference from the classification of one-time versus reversible systems discussed in Section 2.2. The term “reversible” specifically refers to whether a material can return to its original state after undergoing a transformation. In contrast, “dynamic” describes the ability of a system to continuously adapt, evolve, or respond to changing environmental conditions over time, regardless of whether the underlying transformation is reversible. For example, a thermally activated shape-memory polymer that recovers its original shape upon heating represents a reversible system, but it is not necessarily dynamic if the response is pre-programmed and does not adapt to external changes. Conversely, a self-regulating hydrogel that continuously adjusts its swelling behavior in response to pH or ionic fluctuations can be considered dynamic, even if some of its underlying chemical processes are not strictly reversible. This distinction highlights that reversibility is a thermodynamic property of state recovery, whereas dynamic behavior reflects time-dependent adaptability and feedback within the system.

Beyond reversibility, another layer of material behavior lies in whether the programming remains fixed after initiation or continues to evolve. This leads to the distinction between static and dynamic programmability. Static programmability refers to systems where the programmed response is embedded and does not change unless manually reprogrammed. These materials are akin to hardware they follow a defined set of rules and produce consistent outcomes when triggered by specific stimuli. In statically programmed materials, the function is often tied to a single design logic. Shape-memory polymers, once cured with a specific temporary shape, return to a pre-determined configuration upon heating [96,97]. Similarly, pre-patterned dielectric elastomers respond in a predictable fashion to an applied voltage, but the response cannot be altered without modifying the design [98]. Static systems offer robustness and predictability, making them suitable for applications requiring repeatable, reliable performance. However, they lack adaptability a growing demand in environments that require real-time adjustments or evolution of functionality.

In contrast, dynamically programmable materials possess time-dependent or adaptive behavior. These systems can respond not only to initial stimuli but also evolve as conditions change, often exhibiting memory, logic, or feedback capabilities [99]. For example, materials with integrated feedback loops can stiffen or soften based on external mechanical stress, creating bioinspired systems akin to muscles or tendons [100]. Hydrogels that exhibit sequential responses first swelling, then releasing a chemical, then contracting show how time-dependent behavior can be encoded into the material's structure [101]. Yue et al. [102] developed hydrogel systems regulated by nucleic acid–based constitutional dynamic networks (CDNs). By reversibly transitioning between three distinct structural states, the CDNs enabled the hydrogels to switch between high, medium, and low stiffness levels. This tunability was exploited to create self-healing materials, control the release of encapsulated substances in response to stimuli, and regulate enzyme cascade activity in an on-demand manner. In a separate study, Paramanick et al. [103] introduced a robotic platform capable of diverse, active motion behaviors. The robot operated on a differential drive mechanism for locomotion and was equipped with multiple infrared and light sensors. These sensing modules allowed it to detect and respond to environmental cues, such as nearby obstacles and changes in ambient light, enabling precise spatiotemporal navigation and control.

Dynamic programmability often requires multi-layered architecture, incorporating sensors, responsive networks, and sometimes embedded computation. These materials are central to soft robotics, where limbs and grippers adjust in real time to irregular surfaces, or in bioengineered tissues that remodel in response to mechanical cues. The growing integration of digital programming with physical matter, sometimes referred to as "material computing," allows even more complex instructions to be embedded into dynamic systems. The main advantage of dynamic programmability is its potential for autonomy and intelligence [104]. Materials no longer act simply as passive responders, but as agents capable of learning and adapting, similar to biological tissues. This opens the door to next-generation applications in wearables, responsive architecture, and adaptive prosthetics, where real-time, context-aware behavior is critical. However, the complexity of such systems demands new paradigms in modeling, synthesis, and control to ensure reliable performance over multiple cycles and diverse environmental conditions.

## 3 Mechanochemistry as a design tool

Mechanochemistry refers to the branch of science that explores how mechanical forces can initiate or alter chemical reactions. In contrast to traditional thermally or chemically driven reactions, mechanochemical processes occur through the application of force often localized and directional at the molecular or atomic level. This force can distort potential energy surfaces, lower activation energy barriers, and open new reaction pathways that would otherwise be inaccessible under standard conditions [[105], [106], [107]]. Importantly, mechanochemistry does not merely accelerate existing reactions but can fundamentally change the nature and direction of a reaction depending on the applied stress and the molecular environment. The concept of force-activated chemistry is not entirely new it has roots in tribology and materials failure studies, where mechanical stress was often seen as a destructive force [108]. However, in recent decades, this destructive view has shifted toward a more constructive and programmable perspective. Instead of simply breaking bonds, mechanical forces are now used to drive selective chemical transformations, activate specific reaction centers, or even trigger autonomous material responses. This has led to the emergence of mechanochemical design as a deliberate strategy in materials engineering, where forces are applied not to damage materials, but to functionally control their properties [109,110].

The appeal of mechanochemistry lies in its unique ability to provide spatiotemporal control over chemical reactions. Force can be applied precisely when and where a reaction is needed, often without requiring heat, solvents, or external reagents. This opens the door to greener, cleaner, and more energy-efficient material transformations. In programmable materials, mechanochemistry offers a direct interface between physical deformation and chemical function, allowing materials to react in real time to environmental forces just like biological tissues often do. This mechanochemical framework is now being incorporated into the design of stimuli-responsive systems that react to mechanical load in intelligent ways. These include color-changing stress sensors, self-healing polymers that repair upon strain, and adaptive networks that reinforce themselves when damaged [111]. The challenge lies in understanding how to integrate chemical reactivity and mechanical response within the same material, a task that requires careful molecular engineering and a deep understanding of force transmission pathways.

### 3.1 Stress-induced bond activation and molecular rearrangements

**Fig. 4:**
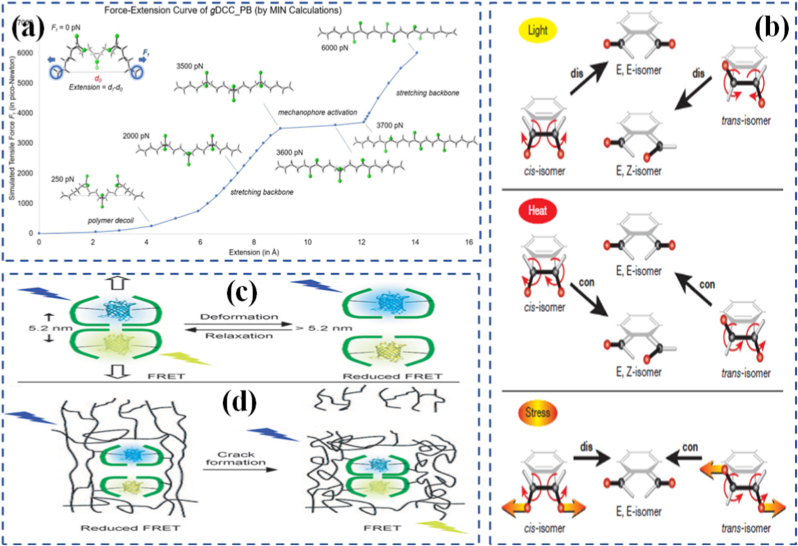
(a) Force-Extension (the distance between the terminal C atoms in Å) curve of *g*DCC-PB trimer generated through the MIN calculation of the FMPES [116], (b) The expected pathways of photochemically activated and thermally activated electrocyclic ring opening of BCB, and the computationally predicted results of mechanically activated ring opening [117], (c) Deformation of the complex increases the distance between the fluorescent proteins, resulting in a reduced FRET process, and (d) In this system, THS is covalently incorporated into a glassy polymeric network [118].

One of the fundamental ideas in mechanochemistry is that mechanical stress can alter the energy landscape of a molecule, making certain bonds more susceptible to breakage or rearrangement. This concept has been extensively studied through the lens of bond potential distortion, where applied force elongates or distorts a bond, effectively lowering the activation barrier for its cleavage or rearrangement. Unlike thermal activation, which distributes energy randomly throughout the system, mechanical activation is directional, meaning it can target specific bonds aligned with the direction of force [[112], [113], [114], [115]]. Stress-induced bond activation typically occurs in polymer backbones or networks under load. When a polymer chain is stretched or compressed, the force is transmitted through covalent bonds, concentrating stress at weak or pre-engineered sites. These "force-sensitive" regions are strategically placed to absorb stress and undergo transformation before the rest of the network fails (Fig. 4 (a)). Depending on the design, the force may trigger bond scission, isomerization, cycloreversion, or even radical formation [116,119]. In reversible systems, the network can reform, while in others, bond cleavage leads to a permanent change in material behavior.

**Fig. 5:**
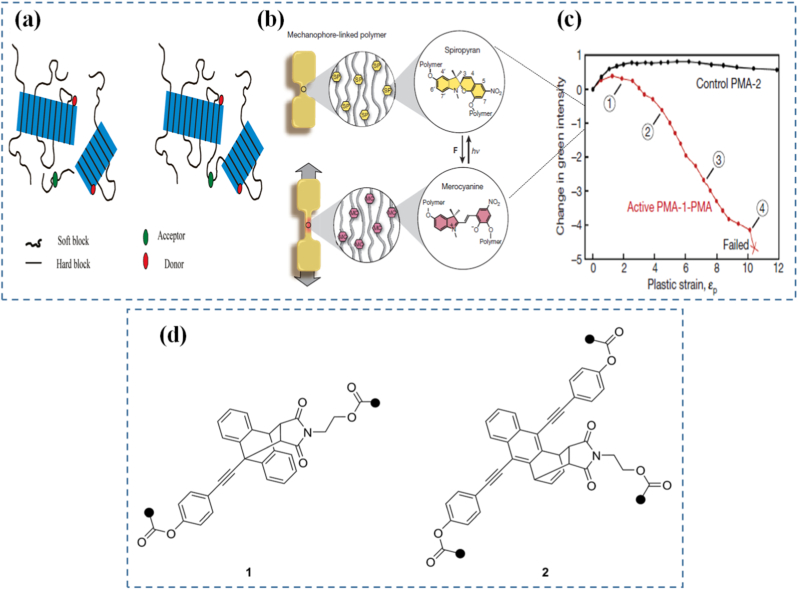
(a) Two kinds of systems studied to probe strain in thermoplastic elastomers (TPE) [123], (b) Schematic diagram of ‘dog bone’ specimens prepared from linear 80 kDa PMA. Upon application of tensile force, a hypothesized conversion between the colourless spiropyran and colored merocyanine forms of the mechanophore occurs, (c) Accumulation of plastic (unrecovered) strain and relative change in green intensity for active PMA-1-PMA and monofunctional PMA-2 control samples after each loading cycle in a fatigue test [124], and (d) Diels-Alder adduct motifs 1 and 2 of p-extended anthracenes studied as mechanophores [125]. (For interpretation of the references to color in this figure legend, the reader is referred to the Web version of this article.)

Molecular rearrangements under force go beyond simple bond breakage. In some cases, applied force can cause geometry shifts, conformational changes, or reorientation of side groups, leading to large-scale structural reconfiguration. For example, rotaxanes and catenanes interlocked molecular architectures can respond to force with controlled sliding or rotation, functioning as molecular switches or machines [[120], [121], [122]]. These rearrangements are not just chemically interesting they translate into macroscale mechanical responses such as stiffening, softening, or shape change. Compared to the strength of interatomic forces, the mechanical forces achievable in materials chemistry offer significant opportunities for mechanochemical transformations. This is particularly evident in polymer sonochemistry, where ultrasound-induced forces can trigger rapid homolytic cleavage of strong covalent bonds, such as C-C bonds, enabling reaction pathways that would otherwise be inaccessible. A notable example is the mechanochemical ring opening of benzocyclobutene (BCB). Hickenboth et al. [117] demonstrated that subjecting BCB-centered polymers to ultrasonication accelerated their electrocyclic ring-opening reactions. When BCB units were tethered to the polymer in a trans configuration, the mechanically induced process enhanced the thermally allowed conrotatory opening. In contrast, with cis linkages, the applied forces redirected the reaction toward the symmetry-forbidden disrotatory pathway (Fig. 4(b)). A fascinating example is the tension-induced unfolding of protein-mimetic materials, where force applied to a folded domain causes it to extend and expose previously hidden chemical groups. This is analogous to how proteins unfold in biological systems under stress, activating sites for catalysis or binding. Clark and colleagues [118] applied Förster resonance energy transfer (FRET) techniques using a protein-based nanosensor to monitor structural deformation within a polymer network (Fig. 4(c and d)). In contrast to the findings of Karthikeyan et al. [123] who observed increased separation between FRET pairs under greater material strain, the Clark group reported the reverse behavior. They embedded FRET pairs inside thermosomes, which were then covalently integrated into a polyacrylamide hydrogel (Fig. 5(a)). During gel formation, internal stresses within the polymer expanded the distance between donor and acceptor, producing a weak FRET signal in the undeformed state. When the hydrogel was stretched or cracked, local stress release allowed the FRET pairs to approach each other, increasing the signal specifically at damaged regions. Materials inspired by this behavior can be programmed to expose reactive functionalities or change surface chemistry under mechanical load, providing a bridge between force sensing and chemical response. Understanding these bond- and structure-level mechanisms is essential for designing programmable materials that respond predictably and selectively to force. Without precise knowledge of how molecules behave under stress, it is impossible to design systems that harness mechanochemistry for useful functions. This is where the integration of experimental and computational tools becomes critical, as discussed in a later subsection.

### 3.2 Role of force-responsive bonds

Mechanophores are a cornerstone of modern mechanochemical design. These specially engineered molecules or molecular subunits are designed to undergo specific chemical changes when exposed to mechanical force. The concept is elegant: incorporate a mechanophore into a polymer backbone or cross-linker, apply force, and induce a predictable and trackable transformation. This transformation can be visual, chemical, mechanical, or electronic, making mechanophores useful as both functional and diagnostic tools. Mechanophores operate by exploiting bond strain sensitivity [[126], [127], [128]]. For example, spiropyrans are one of the most studied mechanophores; when embedded in a polymer and subjected to force, they convert into their colored merocyanine form through a reversible ring-opening reaction. This visible color change can be used to map stress distribution in materials or to signal overstress in real-time [129,130]. Building on related concepts, Nancy R-Sotos et al. [124] utilized the mechanosensitive color change of spiropyrans to visualize stress distribution in solid polymer systems (Fig. 5(b and c)). This mechanochromic response enables real-time mapping of mechanical loads, providing valuable insight for optimizing and reinforcing materials before failure occurs [131]. In another example, Chen's group [132] synthesized a novel mechanophore combining bisrhodamine (Rh) and bisthienylethene (BTE) within poly(methyl acrylate). This dual-functional mechanophore could be activated via ultrasonic forces. The Rh component produced differently colored and fluorescent ring-opening products depending on the force magnitude, while the BTE segment's photochromic switching further modulated the polymer's mechanochromic and mechanofluorescent behaviors. Other mechanophores include Diels-Alder adducts, which undergo retro-cycloaddition under load, or gem-dihalocyclopropanes, which fragment into reactive species capable of initiating further chemistry [[133], [134], [135], [136]]. Göstl and Sijbesma [125] designed mechanophores capable of generating π-extended anthracene derivatives through a mechanochemically induced retro-Diels-Alder reaction of anthracene maleimide adducts (Fig. 5(d)). The resulting extended π-conjugation not only improved fluorescence quantum yield but also shifted the absorption spectrum into the visible region, enabling optical detection under mild conditions. While these examples illustrate how force can activate specific chemical transformations at the molecular level, translating such mechanochemical events into functional materials requires deliberate molecular and structural design strategies, which are discussed in Section 4.

### 3.3 Probing mechanochemical behavior: experimental and computational approaches

**Fig. 6:**
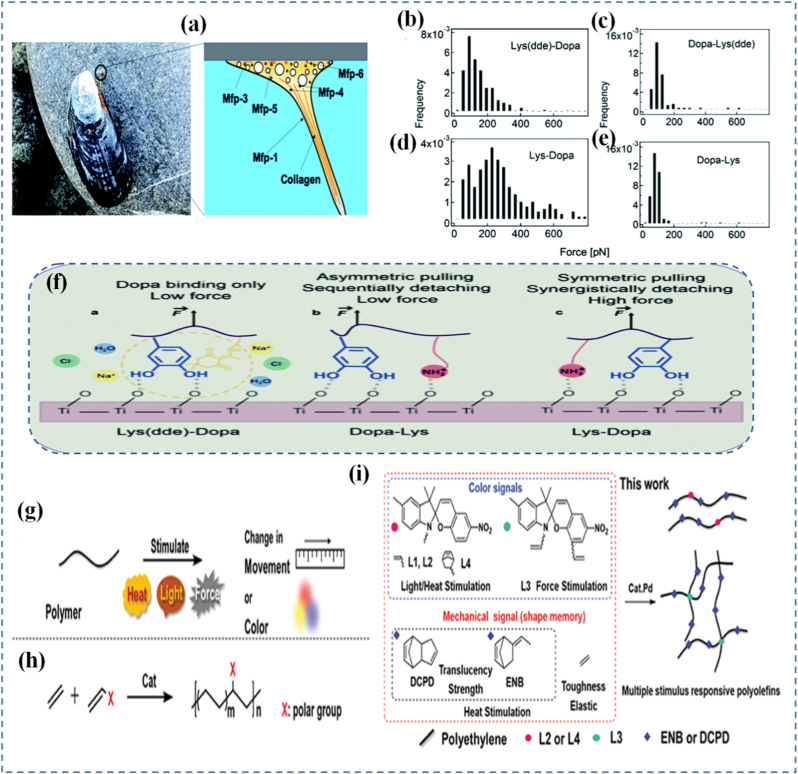
(a) Mussel adhesion and schematic structure of mussel byssus, (b-e) Force distribution of lysine-DOPA and DOPA lysine dipeptides and the TiO_2_ surface, (f) the schematic of the unbinding process of Dopa containing dipeptides [141], (g) Schematic diagram of a polymer's response to stimuli, (h) Transition metal-catalyzed copolymerization of ethylene with polar comonomers, and (i) Preparation of photo-thermochromic and mechanochromic polymers by the direct copolymerization of spiropyran and cyclic monomers with ethylene [143].

Mechanochemistry is inherently molecular, and studying it requires tools that can observe or simulate reactions at the level of individual bonds or molecular motifs. One of the most powerful experimental techniques in this domain is single-molecule force spectroscopy (SMFS) [137]. Using atomic force microscopy (AFM) or optical tweezers, SMFS applies controlled forces to single molecules and measures their mechanical responses [138,139]. This allows researchers to observe bond rupture events, unfolding of protein-like domains, or force-induced isomerizations in real time. At the single-molecule scale, significant work has been done to clarify how mussel-derived proteins achieve strong wet adhesion [140]. In an influential study, Li and co-workers [141,142] applied a “multi-fishhook” single-molecule force spectroscopy strategy to probe the interaction of DOPA residues with different hydrated surfaces. They recorded adhesion forces in the range of 60-90 pN when a protected lysine side chain was present, and remarkably, forces near 300 pN when lysine dipeptides bound to mica (Fig. 6(a–f)). Further investigations by the same group [144,145] into the marine adhesive protein Mfp-5 demonstrated that molecular parameters including the number of binding sites, chain length, and structural arrangement play critical roles in determining both adhesive and cohesive strength. Further translating these molecular-level findings into macroscopic material design provides important guidelines for engineering advanced adhesive systems. The force spectroscopy results on DOPA-containing mussel proteins reveal that strong adhesion arises not only from high individual bond strength but also from the cooperative action of multiple reversible interactions. This has inspired the incorporation of catechol-based chemistries and metal-coordination bonds into synthetic polymer networks to achieve robust yet dynamic adhesion, particularly in wet environments. Moreover, the observed dependence of adhesion strength on parameters such as chain length, number of binding sites, and molecular configuration highlights the importance of controlling polymer architecture and functional group density in bulk materials. Another key insight is the role of sacrificial bonds and dynamic cross-links in dissipating mechanical energy, which has been widely adopted in the design of tough, fatigue-resistant adhesives. Additionally, the ability of these molecular systems to maintain strong interfacial interactions under varying environmental conditions has guided the development of materials with tunable adhesion in response to pH, ionic strength, or hydration state. These examples demonstrate how single-molecule force spectroscopy not only elucidates fundamental adhesion mechanisms but also directly informs the rational design of macroscopic, bioinspired adhesive materials. Single-molecule studies provide critical insights that are difficult to obtain from bulk experiments. For example, SMFS can determine the exact force threshold required to activate a mechanophore, the lifetime of force-activated intermediates, or the rate of reaction under varying force loading rates [146,147]. These measurements can be used to create force reaction profiles, which map how the chemical landscape changes under mechanical input. Such detailed data are invaluable for validating theoretical models and for designing materials with precise activation thresholds.

Complementing experimental techniques are computational tools, especially molecular dynamics (MD) simulations. MD allows for the simulation of how molecules behave under applied stress, providing a time-resolved picture of bond deformation, energy redistribution, and reaction initiation [[148], [149], [150]]. Huo et al. [151] demonstrated the capability of MD simulations to reveal detailed molecular-scale mechanisms in polymer systems. They investigated a bidisperse triblock copolymer elastomer containing spiropyran mechanophores, systematically placing these mechanophores on the shorter chains, the longer chains, or both. The study showed that positioning mechanophores on the short chains resulted in quicker activation and greater activation efficiency, whereas long-chain incorporation produced a slower response. MD simulation results attributed this behavior to microphase separation and the anchoring of midblock ends at the glassy-rubbery interface. Under tensile deformation, short chains were found to extend and align more readily in the loading direction, as reflected by a pronounced increase in their end-to-end distance. Specialized variants like steered molecular dynamics (SMD) can mimic SMFS experiments in silico, helping to predict mechanophore behavior or identify new force-sensitive motifs [152]. In a recent study, the folding pattern of a single polymer chain within its single crystal was investigated to gain deeper insight into the crystallization mechanism [153]. By employing a combination of AFM-based SMFS and SMD simulations, the researchers examined the chain-folding behavior of polyethylene oxide (PEO) within its single crystal. Their findings revealed that in crystals formed from dilute solution, the PEO chains adopted an adjacent re-entry folding pattern. In contrast, crystals obtained from the melt exhibited nonadjacent folding with large and irregular loop structures, which resulted in pronounced force fluctuations in the force-extension profiles. This integrated experimental and computational approach offers a powerful strategy for directly probing the chain-folding configurations of polymer single crystals at the single-molecule level. In a recent study, researchers explored an innovative antiviral strategy involving DNA aptamers as potential inhibitors targeting the spike protein of emerging SARS-CoV-2 variants, including Omicron and JN.1 [154]. Recognizing the limitations of vaccine efficacy against rapidly evolving variants, the study focused on a specifically engineered DNA aptamer, AM032-4, known for its high specificity and binding affinity. Using SMD simulations, the authors investigated the interaction between AM032-4 and the receptor-binding domain (RBD) of the spike protein. Their computational analysis provided detailed insights into the binding mechanisms, revealing that the aptamer maintained strong and stable interactions with the RBD, even amidst sequence variations. This work highlights the potential of DNA aptamers as adaptable antiviral agents and demonstrates the utility of SMD simulations as a powerful tool for characterizing aptamer–protein interactions at the molecular level.

These simulations also help bridge the gap between molecular behavior and macroscopic properties. By linking atomic-level events to changes in polymer conformation or network structure, MD simulations inform multiscale models that can predict how an entire material will respond under load [155,156]. When combined with finite element analysis or continuum mechanics, these tools allow researchers to explore structure property relationships across scales, guiding the rational design of programmable materials. In a study by Park et al. [157], the thermo-mechanical behavior of shape-memory polyurethane (SMPU)-silica nanocomposites was investigated using a coarse-grained (CG) MD model to capture atomic-level interactions, with particular attention to the interfacial region between the polymer matrix and silica nanoparticles. The shape-memory performance was evaluated as a function of the hard segment content (HSC) in the SMPU matrix and the nanoparticle weight fraction. The results indicated that higher silica loadings promoted nanoparticle clustering, which impaired shape recovery. Furthermore, the HSC was found to influence matrix–nanoparticle compatibility, thereby affecting the extent of nanoparticle agglomeration and the overall shape-memory properties. These insights were proposed as useful guidelines for designing deformation behaviors in semicrystalline shape-memory polymer nanocomposites and actuators. As these experimental and computational methods continue to improve in resolution and accessibility, they are becoming indispensable for the design, validation, and optimization of mechanochemically active systems. Together, they form the analytical backbone that supports the growing field of mechanochemical material design enabling scientists and engineers to push the boundaries of what programmable matter can do.

## 4 Design of force-responsive polymers

Force-responsive polymers are engineered macromolecules capable of translating mechanical stress into predictable chemical or physical changes. At the heart of their design lies the integration of molecular motifs that respond to applied force by undergoing transformations such as bond scission, isomerization, or conformational rearrangements (Table 2). One of the most promising areas of innovation is the use of dual-pathway responsive mechanophores, such as those developed by Xu et al. [129], where luminescent responses can be triggered via distinct force thresholds. This concept allows for tunable sensitivity and multifunctional response modes. Additionally, mechanical gating is emerging as a precise control strategy, enabling the release of chemical payloads or the initiation of reactions only under mechanical force, as reviewed by Versaw et al. [163].These polymers are often embedded with mechanophores molecular units that undergo a force-induced reaction allowing the polymer to function as a stress sensor, actuator, or damage reporter. Mechanophores are not limited to a single reaction type. Some, such as Diels-Alder adducts, undergo force-induced cycloreversion, enabling reversible stress response mechanisms [164]. Others exploit selective bond scission, with their behavior determined by both the chemical identity of the mechanophore and the surrounding polymer matrix. The activation force required to trigger mechanophore reactions is critical to their practical utility. The versatility of polymer chemistry provides a wide canvas for tuning these behaviors, from modifying chain flexibility to incorporating dynamic covalent bonds [75]. The mechanical response of these polymers depends on a balance between macroscopic force application and localized molecular deformation. Engineering these systems involves careful control of polymer architecture, such as block copolymer arrangements or crosslinking density, to direct mechanical force along specific pathways [131,165]. A central principle is the strategic positioning of mechanophores molecular units activated by force at locations like chain centers or crosslinking junctions to maximize efficiency under stress. This concept was foundationally demonstrated by Davis et al. [124], where covalent bond rupture in response to mechanical force was leveraged to initiate chemical transformations. Applications of force-responsive polymers span from self-reporting materials that change color upon damage to self-healing systems where stress triggers chemical repair. One such example is polyolefins that simultaneously respond to light, heat, and force to change color or structure (Fig. 6(g–i)) [143]. In biomedical contexts, they are explored for targeted drug delivery systems that respond to the mechanical environment of tissues. Ma et al. [166] reviewed various mechanical force-responsive drug delivery systems that are designed to release drugs only in strained or stressed environments critical in cancer therapy or responsive implants. This complements earlier work on stress-triggered drug delivery systems architectures [167]. In soft robotics, such materials offer potential for smart skins and structural components that sense or adapt to force in real-time. The integration of mechanochemistry into additive manufacturing platforms and intelligent skins is reviewed extensively by Ghanem et al. [131], who emphasize their utility in real-time adaptive structures. Challenges remain in optimizing the force sensitivity and fatigue resistance of these polymers. Mechanophore activation thresholds must align with real-world force magnitudes, and repeated cycles of stress can lead to irreversible degradation if not carefully engineered. Willis-Fox et al. [168] discussed scale-up strategies and highlighted the role of polymer architecture, particularly supramolecular and modular assemblies, in optimizing durability and reusability.

**Table 2:** This table will compare different classes of mechanically-driven materials based on their design principle, stimuli type, actuation mechanism, and key application area.

Material System	Stimuli Type	Actuation Mechanism	Structural Principle	Applications	Ref.
Force-responsive polymers	Mechanical stress	Bond scission or rearrangement	Mechanophore integration	Stress mapping, damage sensing	[158]
Kirigami-based materials	Stretching, bending	Controlled buckling, opening/closing cuts	Origami/kirigami geometric patterning	Flexible electronics, soft robotics	[159]
Programmable hydrogels	Swelling, deformation	Volume expansion/contraction	Cross-linked polymer networks	Drug delivery, shape morphing devices	[160]
Strain-tuned microstructures	Tensile/compressive	Phase shift or lattice distortion	Engineered microstructure design	Sensors, energy absorption	[161]
Elastomeric composites	External load	Elastic reconfiguration and stress redistribution	Soft matrix with embedded particles	Artificial muscles, adaptive skins	[162]

### 4.1 Molecular design of force-responsive polymers: from mechanophores to functional materials

**Fig. 7:**
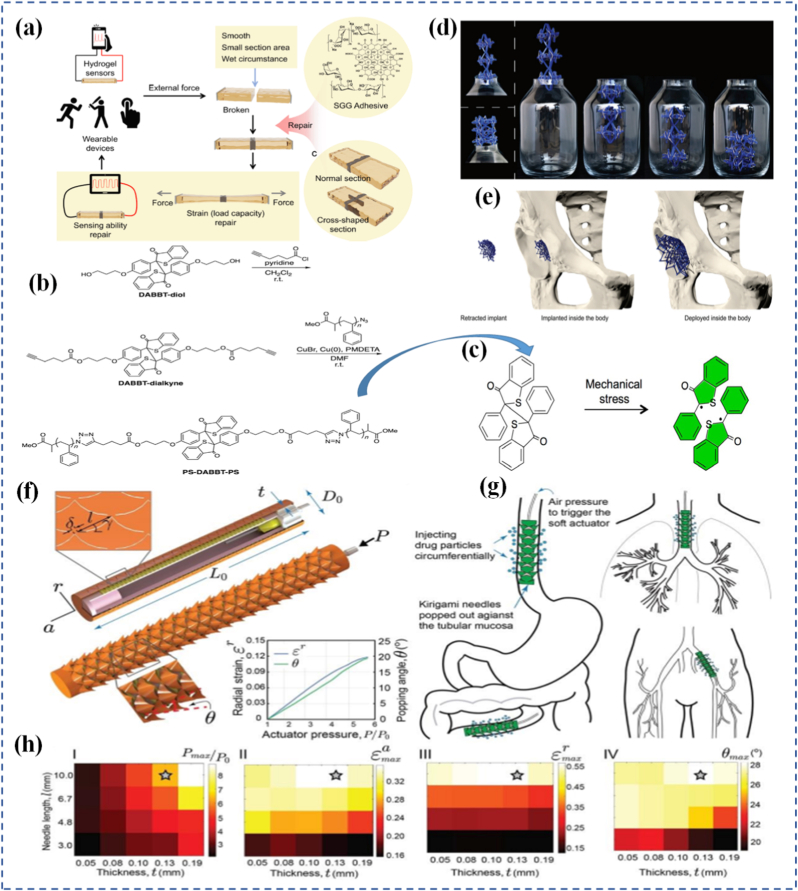
(a) Repair strategy for hydrogel sensor [141], (b) Synthesis of DABBT-dialkyne and PS-DABBT-PS, (c) Chemical structures of DABBT before and after applying mechanical stress [169], (d) A multistable ring-based structure in its compact state fits through the bottle opening, while the deployed configuration does not, (e) Multistable structure applied as a bone implant, enabling shape transformation for minimally invasive insertion [170], (f) Schematic illustration of the stent system in the undeformed and deployed configurations, consisting of the pneumatic soft actuator integrated with a kirigami shell, (g) Schematic of deployment of the injectable kirigami-based stent in the tubular segments of the GI tract, trachea, and iliac artery, and (h) A systematic study to predict the effect of *t* and *l* on the evolution of *ε*_*a*_, *ε*_*r*_, and *θ* as a function of the applied actuator pressure (*P*⁄*P*_0_) for an esophageal-sized stent [171].

The utility of mechanophores goes beyond visualization. In self-healing materials, mechanophore activation can release healing agents or initiate crosslinking reactions at damaged sites (Fig. 7 (a)) [172]. In stress-adaptive systems, force-triggered reactions can locally stiffen a material to prevent further deformation. The diversity of mechanophore chemistry allows for tailoring response thresholds, reversibility, and functionality to suit different applications ranging from aerospace components to soft tissues [173,174]. A particularly exciting area is the programming of mechanophores to exhibit multiple functions. In a separate study, Craig and co-workers [175] reported a double-network hydrogel incorporating a “gated” mechanophore formed by combining a 2-methoxy-gem-dichlorocyclopropane mechanoacid with a methyl methoxycyclobutene carboxylate unit. Incorporated into both linear and crosslinked polymers via radical copolymerization, this design significantly increased internal solution acidity by roughly an order of magnitude when stretched. Upon release of the applied force, the hydrogel recovered its original shape. Ultrasonication confirmed the mechanochemical liberation of HCl, while single-molecule force spectroscopy demonstrated enhanced toughness of the polymer chains. This multifunctional mechanophore system yielded a robust, thermally stable hydrogel capable of releasing acid in response to mechanical strain. For example, a single force-activated molecule might change color, emit light, and generate a reactive intermediate simultaneously. In a notable study, researchers demonstrated that force-reversible C-N bonds formed through click chemistry between triazolinedione (TAD) and indole derivatives can be effectively utilized for molecular-level engineering of mechanically responsive materials [176]. The TAD-indole adducts served as crosslinking points within dry-state covalently cross-linked polymers, enabling real-time, reversible stress-responsiveness at ambient temperature. Although the TAD-indole reaction is exergonic and forms stable adducts under standard conditions, the study revealed that these adducts could dissociate under mechanical stretching when embedded within a polymer network, subsequently reverting to their original components. Remarkably, the TAD moiety was shown to recombine spontaneously with indole units after bond dissociation, thereby facilitating the dynamic adjustment of polymer segment conformations and preserving network integrity. This approach offers a generalizable strategy for designing tough, covalently cross-linked polymer systems with simultaneous improvements in mechanical strength and ductility an outcome that is typically difficult to achieve using conventional chemical methods. Researchers in the Otsuka group [169] have introduced a family of cleavable mechanophores that, upon force-triggered bond scission, yield pairs of persistent, vividly colored free radicals. A representative example is diarylbibenzothiophenone (DABBT) (Fig. 7(b and c)), which undergoes a reversible mechanical dissociation process. The stability and lifetime of the resulting radicals are highly dependent on the surrounding medium. By combining several radical-type mechanophores each capable of producing distinct colors such as pink, green, or blue polymers can be engineered to display a broad spectrum of hues upon mechanical activation. This multifunctionality enables the creation of "intelligent" materials that not only sense and respond but also log their history or report damage through built-in memory functions. To be effective in materials design, mechanophores must be carefully positioned and oriented within the host matrix to ensure efficient force transmission. This often involves copolymerization, surface grafting, or supramolecular assembly. The integration of mechanophores into real-world systems is an engineering challenge, but one that has seen significant progress due to advances in synthetic chemistry, computational modeling, and nanoscale characterization.

**Fig. 8:**
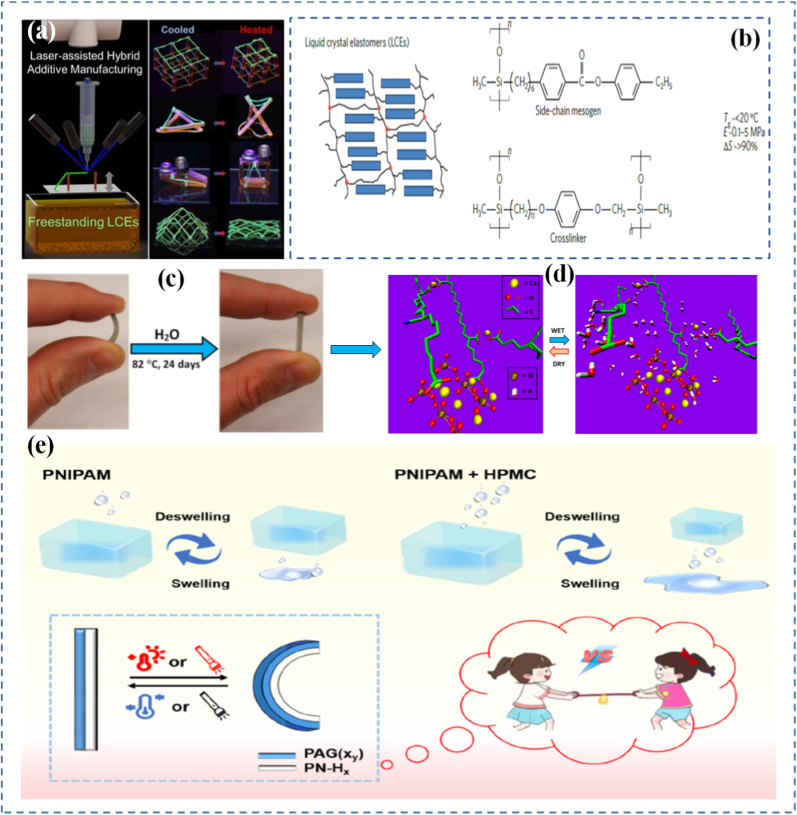
(a) Hybrid 4D printing combining laser-assisted DIW and DLP enables one-step fabrication of complex LCE-based active architectures [186], (b) LCEs are a subclass of crystal polymer networks for which the polymer backbone is commonly polysiloxane and the crosslink density is low [187], (c) Illustration of mechanoadaptability, showing transition from soft and flexible to hard and brittle states, (d) Water-responsive ionic interactions between polymeric carboxylate anions and inorganic cations, including free Ca^2+^ or surface-bound cations on C-S-H particles [188], and (e) Driving mechanism of the Janus hydrogels [189].

Mechanical programming through origami and kirigami provides a geometrical route to encode complex shape transformations in thin-sheet materials. Origami relies on folding, while kirigami incorporates both folding and cutting, allowing for more extensive reconfigurability. These ancient arts are now repurposed in the design of materials that can deform in programmable ways under external forces. By embedding fold patterns into materials, designers can induce predictable, reversible 3D shape changes from 2D sheets [[177], [178], [179], [180]]. At the molecular level, origami/kirigami-inspired designs have been implemented through microfabrication and lithography, enabling integration into materials like polymer films, metallic sheets, or hydrogels. This is especially evident in Ning et al. [181], where lithographic techniques were used to create 2D sheets that fold into functional 3D structures upon exposure to heat or moisture. These structures translate simple mechanical inputs into amplified motions, offering high degrees of freedom in how a material stretches, bends, or twists. This geometrical strategy is especially valuable for systems where active control (e.g., electronic or chemical) is limited or undesirable. These designs have found applications in deployable structures, wearable devices, and soft robotics, where compactness, mechanical adaptability, and multifunctionality are needed. Jin and Yang [182] demonstrate how kirigami structures simplify actuation and control in soft robotic frameworks through pop-up features that enable multiple degrees of motion. Similarly, Ai et al. [183] explore creased patterning in origami/kirigami materials to create precise, lightweight robotic components capable of navigating complex biological spaces. In biological systems, origami-inspired scaffolds mimic tissue folding or organ morphogenesis, opening possibilities in regenerative medicine. In Fig. 7(d and e), multistable structures demonstrate how compact-to-deployed transformations enable passage through confined spaces and potential use as dynamic biomedical implants [170]. This aligns with origami-inspired strategies for navigating complex biological environments and mimicking tissue morphogenesis. Wu et al. [184] reveal how such structures enable untethered soft robots to move within the gastrointestinal tract for potential drug delivery or surgical assistance. These compact, foldable devices amplify small mechanical inputs into targeted actions, offering a non-invasive route for internal diagnostics and therapy. Likewise, Babaee et al. [171] emphasize kirigami's role in healthcare from morphing tissue scaffolds to stretchable biosensors. This concept is further extended in Fig. 7 (f, g) and Fig. 7 (h), where a kirigami-integrated pneumatic soft actuator is shown in both undeformed and deployed configurations as part of a stent system. The injectable stent can be deployed in tubular segments such as the GI tract, trachea, and iliac artery, with a systematic study (Fig. 7(h)) predicting how design parameters affect strain and angular deformation under varying actuator pressures, specifically for esophageal applications. Hybrid designs that combine origami/kirigami with active materials such as light-responsive liquid crystal elastomers (LCEs) are also gaining traction. Leanza et al. [185] describe LCE origami constructs that change shape thermally or optically, enabling reconfigurable structures that adapt autonomously to environmental cues. Ultimately, these mechanically programmed materials enhance durability, compactness, and adaptability, making them excellent candidates for wearable electronics, deployable solar panels, or regenerative scaffolds. In Fig. 8(a), Peng et al. [186] fabricated LCE fibers on a square-shaped adaptable framework. When subjected to mechanical loading, the framework transformed into a parallelogram-like configuration, which could revert to its original square shape upon heating the LCE fibers. Their study also showcased the deployment of a 3D-printed tensegrity model and the shape reconfiguration of an LCE-based lattice pyramid. Unlike LCE sheets, LCE fibers or strips offer the added benefit of localized activation such as through Joule heating allowing for more intricate and programmable folding sequences. With advances in computational origami improving predictability and design optimization, the future of programmable matter rooted in ancient geometry is unfolding rapidly.

### 4.2 Programmable hydrogels and elastomers

Hydrogels and elastomers serve as excellent platforms for programmable mechanics due to their flexibility, water content, and ability to host stimuli-responsive chemistries. Hydrogels, which are networks of hydrophilic polymers, can undergo significant volume changes in response to environmental cues such as pH, temperature, or mechanical force [190,191]. Elastomers, on the other hand, offer elastic deformation over large strains and are widely used in soft actuators, stretchable electronics, and biomimetic devices [[192], [193], [194]]. One of the most established strategies involves using dual-network hydrogels, where one polymer network absorbs strain through plastic deformation and the other maintains mechanical integrity. This results in materials with strain-hardening behavior and enhanced resilience, as reviewed by Jiao et al. [195], who demonstrated such programmable morphing hydrogels in soft actuators and biomimetic robots. Recent developments include the creation of logic-encoded hydrogels that deform in specific patterns upon stretching or compression, enabling applications in shape morphing and soft computation. Light-sensitive or magnetically driven gels are also emerging, where mechanical response is indirectly programmed via external stimuli. For example, White and Broer [187] discuss how LCEs (Fig. 8(b)) can be programmed to localize mechanical response, forming reversible surface features. Similarly, Dolui et al. [162] and Musso [188] highlight the challenges and emerging designs of mechanoadaptive elastomers, materials that stiffen or soften in response to mechanical load, mimicking biological tissues. When a small sample is pressed between two fingers, it bends easily. However, after exposure to water, its stiffness increases so significantly that it can no longer be bent under the same or even greater pressure (Fig. 8(c and d). Such multifunctionality makes them attractive for applications in tissue engineering, drug delivery, and microfluidic systems. One exciting direction is the integration of feedback loops into these materials, creating systems that can self-regulate their mechanical behavior. Liu et al. [196] developed hydrogel scaffolds that deform in time-controlled ways and respond to swelling by stiffening critical for minimally invasive medical implants. Similarly, Wang et al. [197] created polymorphic hydrogel fibers capable of extreme mechanical programming via macromolecular conformational control. Soft computation is an emerging frontier, where the deformation of hydrogels or elastomers can encode logic or actuation. Wang et al. [189] introduced Janus hydrogel actuators with time-programmable behavior mimicking neural-like temporal control in artificial systems (Fig. 8(e)). While such complexity increases design challenges, it also opens the door to materials that behave more like living tissues or artificial muscles.

### 4.3 Tuning microstructure through strain

**Fig. 9:**
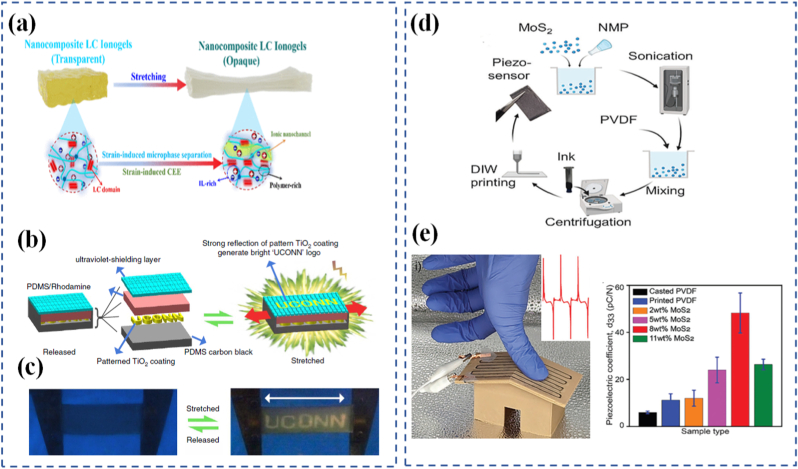
(a) Schematic illustration of the preparation process of the ionogel with straininduced microphase separation [199], (b) Design strategy and mechanical responsive encryption properties of the encryption mechanochromism, (c) The hidden ‘UCONN’ logo concealed at released state and revealed upon being stretched to 17% strain with excellent reversibility [200], (d) Ink formulation and 3D printing process of the PVDF and PVDF-MoS_2_ nanocomposite, and (e) Measurements of the piezoelectric coefficient [201].

Strain-induced changes in microstructure provide another avenue for mechanical programmability. When mechanical forces are applied to materials with hierarchical or anisotropic internal structures, these microstructures can realign, fracture, or reconfigure, leading to macroscopically observable changes in properties. In many polymeric systems, this effect is leveraged to create strain-responsive optical or conductive changes, allowing materials to sense and respond to mechanical deformation. Lee et al. [198] designed a shape-reconfigurable strain sensor where strain-induced stress concentrates along a patterned structure, enabling ultrahigh and tunable sensitivity. Similarly, ionogels with anisotropic nanostructures exhibit strain-induced phase separation (Fig. 9 (a)), modulating their ionic conductivity in response to deformation a strategy well-executed by Li et al. [199]. In optical systems, strain can alter microstructure to dynamically tune photonic properties. Lin et al. [202] developed a biomimetic photonic elastomer that forms wrinkle-lattice patterns under mechanical and moisture stimuli, enabling reversible deformation tracking and optical memory storage. This is echoed in Zeng et al. [200], who describe bio-inspired mechanochromic materials using strain-dependent cracks to produce reversible color changes ideal for self-reporting structural damage or wearable displays (Fig. 9(b and c)). Strain can also modify electrical properties. For example, spin-coated polyaniline thin films exhibit piezoresistive behavior due to the disruption of their highly ordered microstructure during deformation, making them useful for resistive strain sensing [203]. Additionally, Islam et al. [201] showed that shear-induced alignment in 3D-printed PVDF-MoS_2_ composites enhances piezoelectric sensitivity, enabling the fabrication of high-performance conformal sensors. As shown in Fig. 9(d and e), the tailored ink formulation and 3D printing process allow precise deposition of PVDF and PVDF-MoS_2_ nanocomposites, while measurements of the piezoelectric coefficient confirm the improved electromechanical performance.

This phenomenon is particularly important in nanocomposites and structured films, where particles or domains are distributed within a soft matrix. Under strain, these inclusions may align, form conductive pathways, or change their spatial distribution, resulting in altered thermal, electrical, or mechanical performance. In nanocomposite hydrogels, the incorporation of conductive particles like magnetite nanoparticles or MXene nanosheets enables strain-sensitive electrical responses. For instance, Li et al. [204] developed ionic-covalent hydrogels grafted with magnetite nanoparticles that become electrically conductive under mechanical deformation, making them ideal for sensitive strain sensors. Similarly, Liu et al. [205] created a healable strain sensor using a biomimetic polyurethane-MXene composite that retains flexibility and repairability two traits essential for wearables and soft robotics.

The concept of tunable microstructure also extends to biomimetic materials. Lavrador et al. [206] demonstrated how nanocomposite hydrogels can dynamically tune their mechanical properties under strain, replicating load-bearing functions seen in tendons or ligaments. Pan et al. [207] extended this to wearable sensors with biomimetic structures that optimize mechanical strength and stretchability while maintaining strain-sensing performance. In another bioinspired example, Guo et al. [208] designed a cephalopod-inspired mechanoluminescent nanocomposite that self-heals and emits light upon mechanical stimulation. This dual functionality strain sensing and visual signaling mirrors natural camouflage and repair mechanisms. The convergence of mechanics and light response also appears in Zeng et al. [209], who describe soft materials with multiscale architectures and dynamic topographies that adapt both their mechanical and optical responses based on input. For example, Almeida et al. [210] used cellulose-based materials to emulate the mechanical behavior of biological tissues while enabling tunable strain responsiveness. These systems achieve selective conductivity, making them candidates for skin-like electronics or prosthetic interfaces. Mimicking these mechanisms through synthetic materials can lead to advanced prosthetics, adaptive load-bearing materials, or even intelligent textiles.

Experimental approaches to study and harness these effects include in situ microscopy under strain, synchrotron X-ray scattering, and digital image correlation techniques. For example, Levitas [211] reviewed in situ approaches used in severe plastic deformation and high-pressure experiments, revealing mechanisms such as phase transitions and strain localization. These real-time methods are particularly useful for tracking microstructure reorganization in polymer blends and nanocomposites. Similarly, Dong and Shin [212] developed predictive models for microstructure evolution during rolling processes in steels, a strategy transferrable to polymer-based materials with multiphase domains. Additionally, Frydrych et al. [213] highlight the growing role of materials informatics, which combines data-driven modeling with high-throughput experiments to accelerate the discovery and optimization of microstructure-responsive systems. Coupling these methods with computational models allows for the prediction and design of microstructure evolution in complex systems. The ability to tune microstructure through strain adds another layer of programmability, turning mechanical inputs into precise and useful outputs.

## 5 Chemically-controlled mechanical response

### 5.1 Covalent and non-covalent chemistries enabling actuation

**Fig. 10:**
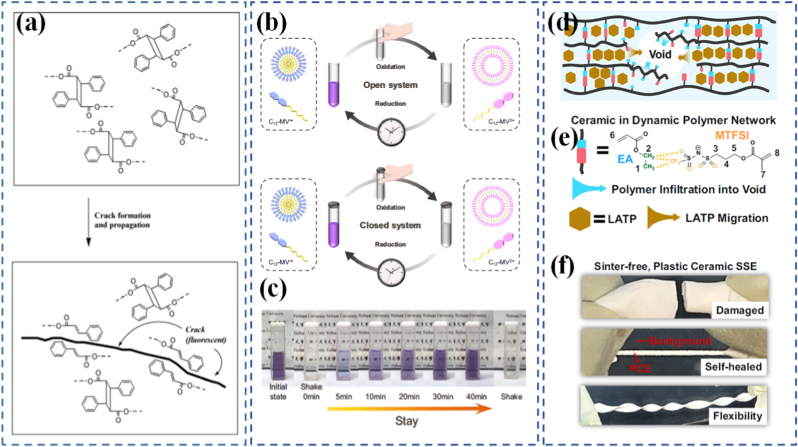
(a) The crack sensing concept [220], (b) Shake-induced out-of-equilibriumredox reaction in an open and closed system, (c) Photos for the solution of the shake-induced transient redox reaction [221], (d) Design concept of the plastic ceramic electrolyte, (e) Embedding the LATP powder into a dynamic polymer network, and (f) Photographs showing the self-healing ability and flexibility of hydrogel [222].

Chemical bonding plays a foundational role in dictating the mechanical behavior of responsive materials. Covalent and non-covalent interactions serve as the molecular levers through which stress, deformation, and movement can be chemically regulated. A foundational perspective is provided by Zarzar and Aizenberg [214], who demonstrate hybrid material designs that incorporate both covalent backbones for structural robustness and non-covalent motifs for reversible responsiveness. This hierarchical organization allows for sophisticated chemomechanical actuation in response to diverse stimuli. Complementing this, Grinthal and Aizenberg [215] emphasize hierarchical chemomechanical feedback as a framework for designing materials that can sense and adapt to their environment dynamically. In covalent systems, the formation and cleavage of bonds result in irreversible or semi-reversible mechanical transformations, enabling functions like folding, unfolding, or network remodeling. These transformations are often triggered by specific chemical cues such as pH changes, redox stimuli, or the presence of catalytic species, which make them ideal for applications requiring programmable or autonomous material behavior [[216], [217], [218]]. Non-covalent interactions, on the other hand, offer a reversible and highly tunable platform for designing responsive materials. Schneider and Strongin [219] explore supramolecular interactions, including hydrogen bonding and π–π stacking, to enable such tunable responses in synthetic polymers. Similarly, Cho et al. [220] demonstrate how specific reversible covalent motifs like cinnamates allow for reversible stress-responsive behavior critical for self-healing or fatigue-resistant systems (Fig. 10(a)). Importantly, the strength and reversibility of these bonds can be fine-tuned, enabling materials that show either rigid mechanical integrity or soft, flowable behavior under different chemical environments [223].

A significant advantage of combining covalent and non-covalent systems lies in the creation of hybrid materials with hierarchical responses. A polymer network can be designed with a covalent backbone for structural support and non-covalent side chains that mediate interactions with stimuli. Upon activation, the non-covalent components can soften or reconfigure, while the covalent structure maintains mechanical stability. Such systems can act as chemical logic gates for mechanical outputs, offering a novel paradigm in responsive material design. For instance, Lang et al. [221] showcase a supramolecular polymerization system activated by mechanical stimuli in closed chemical environments (Fig. 10 (b)). Their design relies on dynamic non-covalent motifs that enable reversible reconfiguration, while the overall polymer matrix ensures stability (Fig. 10(c)). This kind of non-equilibrium polymerization mirrors the chemical logic gating concept central to responsive material frameworks. Similarly, Messing and Schmidt [224] review how hybrid hydrogels combine covalent and non-covalent modules to produce materials with finely tuned mechanical responses and hierarchical organization. They emphasize that such dual bonding strategies allow precise control over local vs. global deformation critical for applications like drug delivery or tissue engineering. He et al. [222] present a high-performance self-healing electrolyte that relies on an aprotic dynamic polymer network (Fig. 10(d and e)). The system demonstrates that introducing non-covalent functionalities like reversible -CH_3_⋯CF_3_ interactions into a covalently cross-linked framework can suppress chemomechanical degradation and extend cycle life in lithium batteries (Fig. 10(f)).

**Fig. 11:**
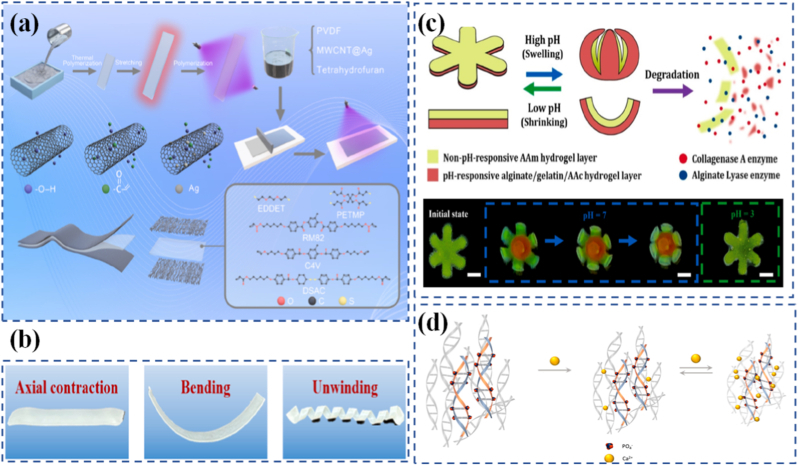
(a) Illustration of the preparation process of the multi-driving mode actuator, (b) Schematic and experimental diagram results of stretching, bending and twisting [228], (c) Schematic illustrating the pH-responsive shape transformation and biodegradation of the bilayer hydrogel gripper [229], and (d) Illustration on mechanism of DNA artificial muscle stimulated by calcium ion (Ca^2+^) [230].

The interplay between chemical bonding and mechanical function is particularly crucial in soft robotics, artificial muscles, and self-healing materials [225,226]. Here, actuation must be both directional and repeatable. Qiu and Zhang [227] report the design of self-healing polymers for soft actuators by combining dynamic disulfide bonds and hydrogen bonds. These bonds confer the dual benefits of mechanical toughness and repeatable actuation an essential feature for robotic applications where directional movement and repeated cycling are required. Further pushing the boundary, Zhang et al. [228] developed soft actuators using disulfide-bonded liquid crystal elastomers capable of multi-mode driving (Fig. 11(a and b)). The materials show reversible mechanical deformation and intrinsic healing through disulfide bond exchange, highlighting the suitability of these chemistries for bioinspired and wearable robotics. Another major advancement comes from Tang et al. [231], who integrated disulfide-based vitrimers into liquid crystal elastomer networks. Their bio-mimetic actuator combines self-healing, photothermal responsiveness, and shape memory demonstrating how synthetic chemistry can engineer oscillatory and feedback-driven mechanical behavior. By drawing from advances in synthetic chemistry and molecular design, researchers are now developing chemomechanical systems that go beyond simple responses to exhibit complex mechanical behaviors, such as oscillatory motion or feedback-driven adaptation. These materials not only blur the boundaries between structural and functional components but also provide a foundation for the next generation of smart, bioinspired, and environmentally adaptive materials.

### 5.2 Stimuli-responsive chemomechanical transformations

Chemical stimuli such as pH, redox potential, and ionic concentration represent powerful means for controlling the mechanical response of materials. These stimuli are particularly attractive because they can be introduced locally, with precise spatiotemporal control, and often occur naturally in biological environments. Responsive materials designed to undergo deformation or phase transition in response to such cues are finding widespread application in biosensors, actuators, and targeted drug delivery systems [232]. pH-responsive materials typically rely on ionizable groups, such as carboxyl, amine, or sulfonic acid moieties, that change their charge state upon protonation or deprotonation [233]. This change alters the internal electrostatic forces, swelling behavior, or hydrogen bonding within the material, leading to significant mechanical responses. Shi et al. [234] demonstrated that hydrogel-based bioactuators, incorporating ionizable groups, can generate reversible mechanical work under physiological conditions, making them ideal for grippers and implantable devices. Similarly, Culver et al. [235] reviewed analyte-responsive hydrogels for biosensing and controlled drug release, highlighting how pH-induced swelling modulates molecular transport and mechanical expansion. Redox-responsive systems also show strong performance. Chen et al. [236] introduced redox-triggered drug delivery systems where the differential redox potential in biological compartments initiates structural changes, aiding in targeted delivery. Dong and Malliaras [237] explored conducting polymers that deform in response to redox cycling, ideal for actuation in implantable or bioelectronic interfaces. A hydrogel containing acidic groups may swell under basic conditions as repulsion between deprotonated groups increases, causing the network to expand and exert force on its environment. Conversely, shrinking occurs when the pH returns to acidic levels. This cyclical behavior is key to designing pH-actuated valves or soft grippers. Kim et al. [229] introduced a biodegradable soft gripper composed of a bilayer structure: one layer made from pH-responsive acrylic acid-containing hydrogels, and the other from non-responsive acrylamide (Fig. 11(c)). This bilayer expands asymmetrically upon exposure to basic pH, mimicking gripping or curling behavior and reverting under acidic conditions ideal for biomedical and transient devices. Yang et al. [238] fabricated a biomimetic hydrogel actuator that undergoes rhythmic deformation through a pH oscillator. Their system achieved over 8 repeated swelling-shrinking cycles with stable gripping behavior, simulating autonomous, and bioinspired soft robotic motion.

Redox-responsive materials often incorporate functional groups like ferrocene, quinone, or disulfide linkages, which can be oxidized or reduced under specific conditions. Such redox changes can trigger either a softening or stiffening of the material by modulating cross-linking density or altering the material's hydration state. These effects are particularly useful in dynamic tissue scaffolds or materials that must adapt to oxidative stress, such as implantable devices. Zou et al. [239] present composite hydrogels enhanced with redox-active and ion-responsive functionalities for wound healing. These systems release exosomes from mesenchymal stem cells in response to ionic or oxidative cues ideal for environments such as diabetic ulcers, where redox gradients fluctuate. The design mimics biological adaptation through metal-ion bridging and oxidative softening. Döring et al. [240] explore rheoreversible hydrogels featuring redox-sensitive groups like disulfide bonds and quinones, enabling reversible changes in cross-linking density. These materials are especially promising in tissue scaffolds that must adapt to oxidative microenvironments during healing or inflammation.

Ionic strength and specific ion concentrations can also act as actuating mechanisms. Ionic-responsive hydrogels, for instance, can undergo sharp volume transitions in response to multivalent cations like Ca^2+^ or Mg^2+^, due to ion bridging or electrostatic screening. Bioinspired ion-sensitive systems are also gaining traction. Kim et al. [230] created calcium ion-responsive DNA hydrogel fibers with enhanced electrochemical conductivity, showing promise for bioelectronic scaffolding and neural interfacing applications (Fig. 11(d)). Such systems bridge biochemical signal transduction and mechanical performance. In other cases, swelling is controlled by osmotic pressure differences, enabling subtle control over pressure-sensitive systems. Sikdar et al. [241] developed smart hydrogels for neural tissue engineering that utilize electrochemically induced redox reactions for shape transformation and molecular release. Their work suggests that redox-active scaffolds can mimic neuromodulatory feedback found in biological tissues. The synergy between ionic and redox responsiveness is further illustrated in Chen et al. [242], where redox-triggered actuation mechanisms are embedded into nanocomposite scaffolds, allowing mechanical modulation without external physical stimuli ideal for minimally invasive therapeutic materials. The integration of ionic response with structural design is especially promising for systems that mimic natural organs or tissues, which often rely on electrochemical gradients for their operation. These chemical stimuli offer not just on/off actuation but graded control over mechanical properties. Through the careful choice of molecular building blocks and environmental conditions, it is possible to fine-tune swelling, stiffness, porosity, or even failure modes, providing materials that behave predictably across a wide range of chemical inputs.

### 5.3 Supramolecular chemistry in load-bearing structures

**Fig. 12:**
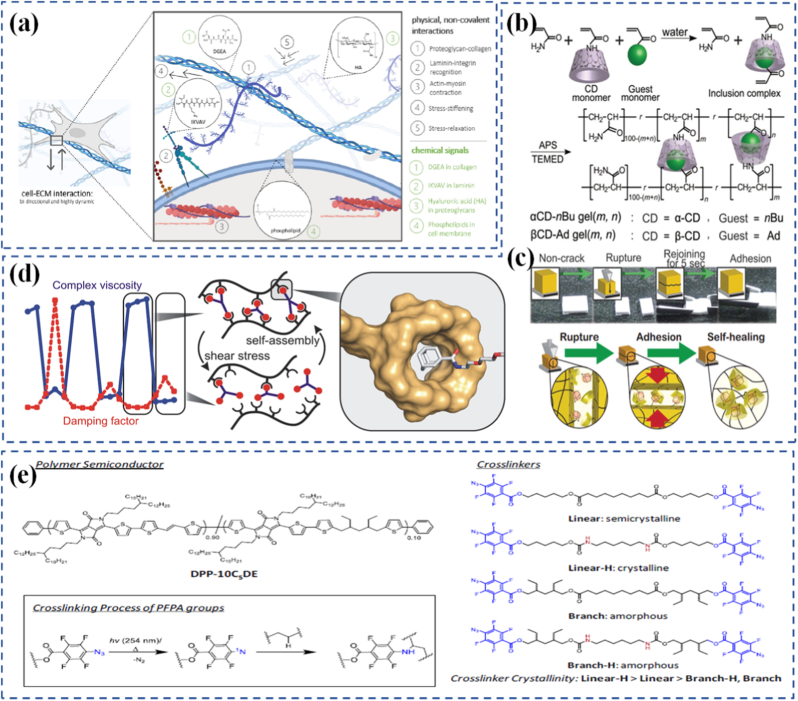
(a) Cell-ECM interactions are bidirectional and highly dynamic [243], (b) Preparation of host–guest supramolecular hydrogels of the β-cyclodextrin- adamantane gel, (c) Schematic illustration of the self-healing between cut surfaces [244], (d) 3D supramolecular network of β-cyclodextrin-hyaluronan and adamantylated cross-linkers enabling tunable stiffness via reversible inclusion complexes [245], and (e) Chemical structure of the four cross-linkers and the DPP-based polymer [246].

Supramolecular chemistry, based on reversible and non-covalent interactions, has become an increasingly important design strategy for creating mechanically robust yet adaptive materials. These systems rely on self-assembly processes that offer both mechanical resilience and dynamic reconfigurability, allowing structures to be load-bearing while still capable of responding to external chemical or mechanical inputs. Rijns et al. [243] present a framework for designing supramolecular hydrogels that replicate extracellular matrix (ECM) complexity through reversible, non-covalent interactions (Fig. 12(a)). These hydrogels integrate with cellular components and adapt to mechanical cues, making them ideal for tissue scaffolding and regenerative medicine. Similarly, their follow-up work [247] emphasizes fully synthetic matrices that mimic cytoskeletal responsiveness using multicomponent self-assembly. This approach closely mirrors the behavior of natural biological systems, such as cytoskeletal filaments and extracellular matrices, which derive their mechanical integrity from hierarchical and dynamic supramolecular interactions.

In engineered systems, supramolecular interactions such as host-guest complexes, metal-ligand coordination, and hydrogen bonding provide the framework for stress distribution and mechanical reinforcement. Polymers containing cyclodextrin or cucurbituril moieties can form inclusion complexes with specific guest molecules, resulting in supramolecular cross-linking. These interactions can be reversibly disrupted or reformed under controlled chemical environments, allowing for on-demand mechanical tuning and self-repair. A foundational review by Yang and Urban [248] details how diverse supramolecular motifs, including cyclodextrins, π–π interactions, and H-bonding, facilitate the reversible assembly of polymers that can autonomously heal under mild conditions. These systems are particularly valuable for soft electronics and coatings. Harada et al. [244] focus on hydrogels assembled via host-guest interactions (especially β-cyclodextrin and adamantane) (Fig. 12(b)). When a cube-shaped β-cyclodextrin- adamantane gel was cut in half, the two pieces instantly rejoined, and the adhesive strength at the joint recovered to 99% of its original value after standing for 24 h (Fig. 12(c)). These networks enable repeated self-healing and tunable modulus changes, making them highly suitable for biomedical applications like injectable scaffolds and wound dressings. A more recent application from Yang et al. [249] demonstrates cucurbituril-modified Fe_3_O_4_ nanoparticles embedded in hydrogels that not only self-heal but also exhibit photothermal responsiveness and stretchability. These magnetically and thermally responsive systems offer multifunctionality for wearable and flexible electronics. Wang et al. [250] describe how integrating macrocycles like cucurbiturils into polymer backbones enables reversible cross-linking that dramatically enhances mechanical integrity without sacrificing responsiveness. Their work highlights supramolecular polymer gels that operate through a combination of hydrogen bonding and host-guest dynamics. Jurtik et al. [245] developed 3D supramolecular networks using β-cyclodextrin-modified hyaluronan and adamantylated cross-linkers (Fig. 12(d)). These materials provide stress-distribution capabilities and adjustable stiffness by reconfiguring inclusion complexes, a mechanism inspired by reversible protein-protein interactions.

One of the key advantages of supramolecular load-bearing materials is their inherent damage tolerance. When a fracture or high strain is applied, these materials can dissipate energy by breaking weak bonds while maintaining overall structural integrity through the rapid reformation of interactions. For instance, Xu et al. [251] developed supramolecular hydrogels engineered with distinct toughening mechanisms that include sacrificial bonds. These materials dissipate energy under strain yet retain structural integrity mimicking the tough-soft combination seen in cartilage and tendons. Similarly, Fang et al. [252] review soft-hard interfaces and emphasize supramolecular strategies as critical for maintaining mechanical function while allowing reversible adaptation ideal for robotic and bioimplant systems. This mechanism mimics the sacrificial bonding strategy observed in biological tissues and opens pathways for materials that are both strong and tough an uncommon combination in traditional polymer systems.

Furthermore, supramolecular systems can be programmed to adapt to specific mechanical profiles by modifying the interaction strength or stoichiometry of the molecular components. This allows researchers to create gradient or anisotropic materials in which mechanical properties vary spatially, depending on the arrangement of supramolecular motifs. Bioinspired anisotropic responses are highlighted in Yang's thesis [253], which discusses multifunctional soft robotic structures built from supramolecular assemblies that show spatially variable swelling/shrinking. This design mimics the cytoskeleton's localized adaptability and enables intelligent robotic gripping and actuation. In a broader systems context, Ye et al. [254] and Wang et al. [255] describe soft robots that use supramolecular architectures such as hydrogen bonding networks or metal-ion coordination for adaptive deformation, self-repair, and load-bearing under dynamic mechanical input. These materials support programmable strain distributions and enhanced fracture toughness. Wu et al. [256] further reinforce the tissue-mimetic angle, reporting highly oriented supramolecular hydrogels capable of guiding cellular alignment and mimicking the anisotropic stiffness gradients of natural tissues. These gels not only support load but also provide directional cues for mechanotransduction. Such strategies are being actively explored in soft robotics, wearable sensors, and tissue-mimetic materials. The ongoing convergence of supramolecular chemistry and polymer science is expected to yield even more versatile and multifunctional load-bearing systems. As computational tools and in situ characterization techniques improve, the rational design of supramolecular architectures will enable responsive structures that not only support loads but also communicate, adapt, and heal like living systems.

### 5.4 Chemical doping and cross-linking to tune mechanical properties

Chemical doping and cross-linking are two well-established yet continually evolving techniques for tailoring the mechanical behavior of polymeric and composite materials. These modifications allow for precise control over stiffness, toughness, elasticity, and actuation behavior, making them essential tools for designing programmable materials. Doping involves the introduction of small molecular or ionic species that alter the electronic or structural environment of the host material, whereas cross-linking refers to the formation of covalent or non-covalent bridges between polymer chains. For example, Chan et al. [257]demonstrated the programming of polymer networks by doping them with primary structural defects, offering a strategy to modulate fracture resistance and elasticity without altering the overall chemical composition. Similarly, Luo et al. [258] engineered super-tough, self-sensing, and shape-programmable polymers using topological cross-linking, including hydrogen bonding and hyperbranched networks, yielding high resilience and dynamic reconfigurability. Cross-linking and doping approaches also enable high-performance actuation. For instance, Yin et al. [259] developed dynamic covalent polymer networks doped with conductive fillers to achieve programmable filler alignment, resulting in tough yet sensitive sensors for human motion monitoring. Photo-crosslinkable systems also benefit from doping strategies. Chen et al. [260] reported dual shape programmability in covalent adaptable networks by combining light-triggered cross-linking and doped nano-additives, enabling both mechanical robustness and reprocessing.

Recent studies illustrate how chemical doping and cross-linking mechanisms control crystallinity, flexibility, and mechanical resilience in conductive polymers and stretchable electronics key enablers of high-performance shape memory and adaptive materials. In conductive polymers, chemical doping directly alters chain packing, crystallinity, and intermolecular interactions, leading to dramatic changes in both electrical and mechanical performance. For instance, Wang et al. [246] demonstrated how doping strategies that modulate cross-linker crystallinity allow fine-tuning of stretchability and conductivity in polymer semiconductors critical for flexible electronics (Fig. 12(e)). Similarly, Zhang et al. [261] showed that doping polymer-grafted gold nanorods into dynamic polymer networks enhances mechanical flexibility while retaining shape memory and self-healing properties. Cross-linking, particularly dynamic and reversible modes, has emerged as the dominant approach for regulating Young's modulus, fracture toughness, and shape memory behavior. A recent review by Zheng et al. [262] explored how dynamic covalent polymer networks based on Diels Alder reactions or boronic esters allow reprocessability, actuation, and enhanced mechanical lifetimes in wearable and robotic systems. Shi et al. [263] further highlighted how supramolecular cross-links in semicrystalline polymers can synergistically enhance mechanical strength and adaptive functionality. The interplay between doping and cross-linking is emphasized in emerging hybrid systems. For instance, Yin et al. [259]created dynamic covalent polymer networks embedded with conductive dopants, resulting in programmable filler alignment, enhanced toughness, and real-time sensing capabilities for human motion. Likewise, Li et al. [264] demonstrated that modulating cross-linking strength and crystallization can achieve the delicate balance of self-healing, toughness, and shape memory all in one polymer platform. Together, these advances show that chemical doping modulates molecular interactions and phase behavior, while cross-linking governs macro-mechanical function.

Recent developments in click chemistry and reversible cross-linking motifs, such as boronate esters or imine bonds, have expanded the design space for mechanically tunable systems [265]. These chemistries allow materials to switch between states for example, from soft to rigid under specific chemical cues. This versatility is especially beneficial in biomedical devices, adaptive clothing, and morphing surfaces that must operate in varying environments. In the field of shape-morphing surfaces, Li et al. [266] demonstrated 3D shape-changing hydrogels that respond simultaneously to temperature and pH. Their composite system enables reversible deformation cycles, showcasing potential for soft actuators or programmable morphing skins. Dual responsiveness is also exemplified in phenylboronic-acid-based hydrogels, which change swelling behavior in response to glucose and pH, as reviewed by Morariu [267]. These materials are under development for biosensors and adaptive biomedical implants. The synergy between doping and cross-linking further enhances design flexibility. For instance, doped and cross-linked hydrogels can show dual responsiveness, exhibiting both pH-triggered swelling and temperature-driven stiffness modulation [268]. These integrated chemistries exemplify how multifunctionality can be embedded into the molecular structure, enabling materials that are not only responsive but also programmable in more than one dimension of performance.

## 6 Multiscale modeling and simulation

### 6.1 Bridging molecular mechanisms to macroscale behavior

Understanding how molecular-scale events translate into macroscopic behavior is a foundational challenge in the field of programmable materials. At the nanoscale, bond formation and breakage, conformational changes, and supramolecular assembly dictate how a material responds to its environment. However, these molecular-level changes often manifest as shape morphing, stiffness modulation, or functional transformation at much larger length scales [269,270]. From a structural and bioinspired standpoint, Lu and Bobrin [271] illustrated how polymeric nanoscale building blocks can be hierarchically assembled to create bulk materials with programmable responses enabled by incorporating nanoscale mechanics into larger-scale predictions. Multiscale modeling serves as a critical tool to connect these realms, enabling researchers to trace the consequences of atomic-level interactions all the way to material-scale performance. This bridging is achieved through hierarchical simulation approaches. Atomistic models, such as MD and quantum mechanical (QM) calculations, provide detailed insights into local bonding environments, energy landscapes, and force transmission pathways [272]. Moon et al. [273] demonstrated a scale-bridging strategy using molecular dynamics to determine the elastic modulus of nanocomposites containing light-sensitive domains, then fed these properties into larger mechanical simulations of deformation under light activation. A related study by Choi et al. [274] further integrated CG models into finite element analyses for predicting macroscopic bending in these systems. CG models then abstract these details into larger units of behavior, which can be fed into mesoscale or continuum mechanics models. These hierarchical layers ensure that the essential chemistry is retained while still making the computations feasible at device-relevant scales [275].

**Fig. 13:**
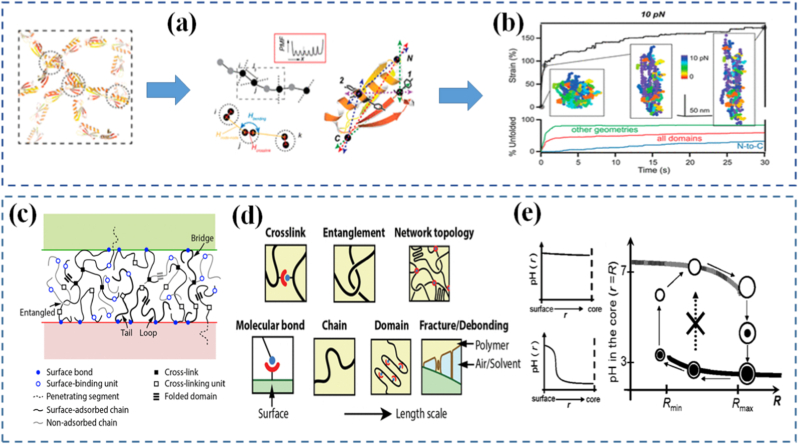
(a) Schematic view of the coarse-graining approach with network optimization to study the mechanical response of protein-based materials made from globular polyproteins, (b) Corse-grained diffusion simulations and experimental comparison of tissue-like materials made from globular polyproteins [276], (c) Schematic illustration of the internal structure of an adhesive polymer film between two solid surfaces, (d) Molecular mechanisms of surface force generation and calibrated bond rupture for simulating delamination and adhesion failures in composites [277], and (e) Operating principle of a chemomechanical oscillator when a pH responsive gel bead is continuously fed by the reactants of a H^+^ autocatalytic reaction [278].

One of the most significant breakthroughs in recent years is the ability to model how molecular conformational changes such as the unfolding of mechanophores or the switching of chemical cross-links can induce strain or stress distributions within bulk materials. These insights enable designers to predict emergent properties, such as how a polymer film might curl, expand, or stiffen in response to localized chemical or mechanical cues. For example, Nowitzke et al. [276] developed a model demonstrating how the mechanical unfolding of protein-based network nodes governs stress propagation in biopolymer gels (Fig. 13(a and b)). Their atomistic modeling of hydrogen bonding and unfolding events accurately predicted macroscopic mechanical behaviors such as toughening and energy dissipation. Similarly, Brighenti, Li, and Vernerey [279] reviewed how conformational switching in mechanophore-containing polymers can be used to encode functional outputs like stiffness modulation and self-reporting fracture. Their model captures how structural reconfigurations at the molecular level dynamically alter bulk elasticity and toughness. Importantly, these models also offer reverse insight, guiding chemists toward molecular motifs that are more likely to produce desired bulk responses.

Furthermore, the success of bridging scales depends heavily on parameter transfer between modeling levels. Accurate force fields derived from lower-level simulations or experiments must be incorporated into higher-level models without loss of fidelity. In a comprehensive review of polymer gels, Xu et al. [280] detailed the implementation of hierarchical cross-linking mechanisms including light- and ion-triggered switches and how these are embedded in multiscale modeling workflows to simulate tunable gel stiffness and shape memory under environmental cues. A crucial component of successful scale-bridging is parameter transfer. In multiscale modeling frameworks, the parameterization of CG force fields plays a central role in enabling this transfer across length scales. These parameters are typically derived either from first-principles calculations, such as density functional theory (DFT), or from experimental data including thermomechanical and structural measurements. Bottom-up approaches map atomistic simulations onto reduced representations by matching structural distributions, force correlations, or free energy landscapes, while top-down approaches fit CG parameters to reproduce macroscopic observables such as elastic moduli, swelling behavior, or diffusion coefficients [281]. When chemical reactions are involved, reactive force fields provide an explicit description of bond breaking and formation with relatively high computational efficiency, making them suitable for large-scale simulations. However, they often rely on predefined functional forms, which may limit their transferability and accuracy for complex chemistries. In contrast, machine learning-based potential functions can achieve near first-principles accuracy by learning high-dimensional potential energy surfaces from quantum mechanical data, although they require large training datasets and may face challenges in extrapolation beyond the training domain [282]. Hybrid approaches that combine physics-based models with machine learning are therefore emerging as promising strategies to balance accuracy, efficiency, and generalizability in reactive material simulations. Raos and Zappone [277] emphasized the use of energy landscapes derived from quantum or molecular simulations to define input parameters in higher-level finite element models. They also explored force calibration during bond rupture to simulate delamination and adhesion failures in composite materials (Fig. 13(c and d)). This demands continuous development of adaptable, multiscale-compatible modeling frameworks that can incorporate dynamic feedback, such as time-varying boundary conditions or nonlinear material behavior, thus faithfully representing real-world conditions. As programmable materials become more sophisticated exhibiting feedback loops, self-regulation, and environmental sensing the need to simulate coupled physical and chemical events across scales becomes increasingly urgent. Bridging these scales not only accelerates the design-to-deployment pipeline but also reduces reliance on expensive or time-consuming empirical trials. As a result, multiscale modeling is emerging as a central pillar in the rational design of future smart materials.

### 6.2 Coupled chemical-mechanical models

In programmable materials, mechanical behavior is often modulated by ongoing chemical reactions or environmental stimuli. Accurately simulating these systems requires models that do not treat chemistry and mechanics as separate domains but rather as dynamically coupled phenomena. Coupled chemical-mechanical modeling aims to address this complexity by integrating reaction kinetics, diffusion processes, and structural mechanics into a unified framework [[283], [284], [285], [286], [287]]. The interplay between mechanical deformation and chemical change is nontrivial. Stress can influence reaction rates through phenomena such as mechanochemistry, while conversely, chemical transformations can alter local stiffness or initiate deformation through swelling, contraction, or phase transitions. Capturing these feedback loops is critical to understanding and predicting material behavior under operational conditions. In their foundational review, Grinthal and Aizenberg [215] emphasized hierarchical chemomechanical feedback as the architectural principle behind responsive materials. They describe how molecular-scale reactions, such as mechanophore activation or dynamic cross-link switching, can propagate stress fields or alter material stiffness an essential insight for applications in soft robotics and adaptive substrates. In biological analogs, Bailles et al. [288] illustrated how mechanochemical coupling drives tissue morphogenesis through delayed reaction-diffusion kinetics and force-induced signaling. These frameworks are now being repurposed for synthetic systems to model processes such as swelling, contraction, and structural reconfiguration. A polymer network embedded with pH-sensitive bonds may show expansion or shrinkage that depends both on chemical exposure and pre-existing stress states. Kaity and Lobo [289] explored emergent shape formation in tissue analogs using reaction-diffusion models coupled with stress-dependent morphogen distribution. Their simulations show how growth and mechanical feedback co-regulate structure a principle directly applicable to shape-morphing soft materials.

One promising approach involves reaction-diffusion-mechanics coupling, in which chemical species diffuse and react through the material while simultaneously influencing, and being influenced by, the evolving stress and strain fields. This type of modeling is especially important for applications like drug delivery systems, responsive gels, and autonomous soft robots, where localized chemical inputs result in complex motion or mechanical output. One foundational contribution is by Weise et al. [290], who proposed a discrete computational model combining mechanical deformation with reaction-diffusion processes. Their work is particularly relevant in contexts where mechano-electrical feedback influences tissue-like behavior, offering a generalized framework for soft robotic or bioinspired systems. In the context of responsive gels, Horváth et al. [278] showed that non-oscillatory chemical reactions could produce oscillatory mechanical behavior, such as self-pulsing or rhythmic expansion (Fig. 13(e)). This is crucial for self-regulating systems like smart drug delivery platforms, where the mechanics evolve based on localized concentrations and feedback. For biologically inspired systems, Belfiore et al. [291] introduced a stress-sensitive Damköhler number to account for how tensile strain modifies intra-tissue chemical reaction rates, particularly in viscoelastic biomaterials. Their model highlights how external loading conditions dynamically interact with reaction kinetics during regeneration or controlled release applications. An experimental model of soft chemical oscillators using iron-catalyzed Belousov-Zhabotinsky gels was introduced by Nava-Medina [292], suggesting pathways for building self-sustained actuation cycles through integrated chemomechanical feedback. To build such models, numerical methods such as finite element or finite difference schemes are typically employed, incorporating constitutive laws that are modified to include chemical terms. These laws account for not only stress-strain relationships but also changes in moduli, cross-linking density, and swelling pressure as functions of chemical concentration. These models often require iterative solution methods and advanced solvers capable of handling nonlinearities and dynamic boundary conditions. Coupled models are increasingly being used not only to predict but also to design and optimize programmable materials. By simulating different chemical pathways, mechanical loadings, and environmental scenarios, designers can identify ideal combinations of material composition and structural design. As these tools mature, they will continue to support the shift from trial-and-error experimentation toward truly predictive, mechanism-driven material development.

### 6.3 Finite element methods (FEM) with chemical inputs

**Fig. 14:**
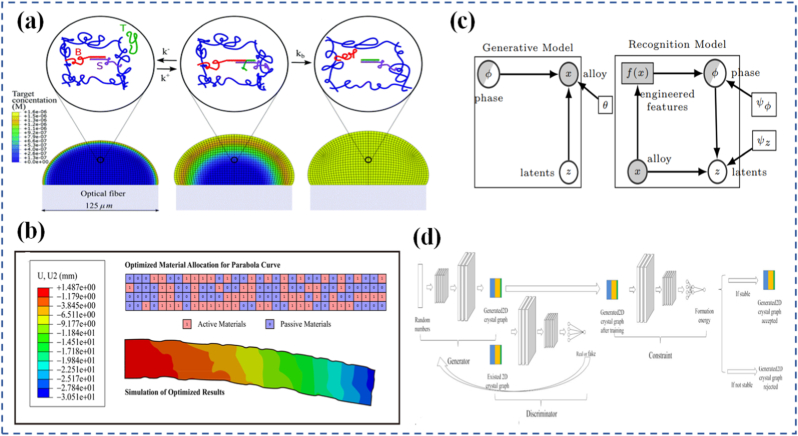
(a) Schematic illustration of the DNA–polyacrylamide hydrogel network and the process of toehold-mediated strand displacement [298], (b) The results for the forward prediction and inverse optimization of case studies [299], (c) Generative (Left) and recognition model (Right) in the disentangled variational autoencoder for inverse design of single-phase high-entropy alloys [300], and (d) Illustration on the DCGAN + constraint model [301].

FEM have long been the workhorse of structural and mechanical analysis, particularly in civil engineering, automotive design, and biomechanics. In the context of programmable materials, FEM is being extended beyond its traditional role to include chemically active domains. This expansion allows researchers to model not only the distribution of stress and strain, but also how these distributions evolve in response to stimuli such as pH changes, redox reactions, or photochemical transformations [[293], [294], [295]]. To incorporate chemical responsiveness into FEM simulations, the governing equations are augmented with chemical transport and reaction kinetics modules. In a hydrogel that swells in response to ionic stimuli, the FEM simulation can account for the changing volume fractions of solvent and polymer, local ion concentration, and osmotic pressure. For example, Vernerey et al. [296] provide a comprehensive review of hydrogel modeling, including formulations for osmotic pressure, solvent diffusion, and reaction rates within cell-laden 3D matrices. This work bridges bio-chemo-mechanical interactions. Askari-Sedeh and Baghani [297] explore a fully coupled 3D FEM model where pH-driven ion transport and swelling are considered under mechanical constraints like torsion and internal pressure, showcasing applications in soft robotics. Jonášová et al. [298] present a finite element implementation that combines reaction-diffusion processes with swelling and mechanical constraints (Fig. 14(a)). Their model includes kinetics of branch migration and simulates the dynamic evolution of hydrogel structure under stimuli-responsive conditions. Yu et al. [302] introduce a mixed hybrid FEM framework that simultaneously considers mechanical deformation, solvent flux, and ionic transport. Their three-field formulation captures the coupling between ionic concentration, swelling pressure, and mechanical stress, critical for designing bioresponsive devices. These chemical inputs are translated into mechanical consequences through modified constitutive models, which may describe non-linear elasticity, viscoelasticity, or plasticity with parameters that evolve over time and space.

Implementing such simulations requires detailed material characterization, particularly with respect to how chemical composition influences mechanical properties such as modulus, yield stress, and failure strain. Experimental data from techniques like rheometry, spectroscopy, and swelling tests can provide the necessary input functions. These functions are then coded into FEM software either through custom user-defined material models or built-in modules, depending on the complexity of the desired behavior. For instance, Yang et al. [303] developed a user-defined constitutive model for hydrogel adhesives by correlating polymer entanglement physics and contraction mechanics with adhesion behavior. Experimental input from swelling and mechanical testing informed the FEM parameters governing time-dependent contraction and failure. Kang et al. [304] implemented a custom material subroutine in FEM to simulate free swelling and equilibrium behavior of hydrogels. Their formulation included chemical inputs affecting internal stress, tied directly to thermodynamic swelling pressure and deformation ideal for modeling plant-inspired actuation systems. Another application of FEM with chemical inputs lies in simulating heterogeneous structures such as gradient hydrogels or layered composites, where different regions respond to stimuli in distinct ways. Barreto [305] further emphasizes how rheological parameters extracted from live cells can be integrated into FEM simulations for biomechanical predictions. This concept extends to smart hydrogels, especially when cellular traction or structural gradients are involved. This allows for predictive modeling of shape changes, crack propagation, and local stress concentrations, helping to preempt failure or design materials that adapt under load. Time-dependent simulations are also critical for capturing transient phenomena like pulsatile swelling or delayed actuation. The integration of chemical inputs into FEM frameworks is helping to create a new generation of simulation tools tailored for intelligent materials [306]. These tools are becoming invaluable not only in research but also in industry, where virtual prototyping of smart sensors, actuators, or biomedical implants can accelerate development and reduce costs. The continued evolution of chemically informed FEM promises to greatly expand our ability to design programmable materials that are both mechanically robust and highly adaptive.

### 6.4 AI-assisted design tools for inverse programming

Inverse design, the process of specifying desired material functions and working backward to determine the necessary structure and composition, represents a transformative shift in materials science. In the field of programmable materials, where the relationship between input stimuli and output behavior is often highly nonlinear and multidimensional, traditional trial-and-error design approaches are no longer sufficient. AI, particularly ML techniques, is now being deployed to accelerate and optimize this inverse programming process. One recent advancement is by Jin et al. [299], who integrated ML into the inverse design of 4D printed structures, enabling the prediction of material allocation based on desired shape changes under stimuli (Fig. 14(b)). Their method jointly solves both forward and inverse design problems, dramatically accelerating material selection and spatial patterning. In a broader context, Wang et al. [307] review how ML models have been tailored for material design tasks, especially when linking microscale properties with macroscale performance through multiscale learning and inverse mapping. Sun et al. [308] showcase a ML-enabled framework for both forward prediction and inverse design of 4D-printed active plates, addressing geometric and material nonlinearity. Their results demonstrate how AI accelerates optimization cycles that would otherwise be computationally expensive. Deep learning is further explored in Zheng et al. [309], who applied generative networks for mechanical metamaterials, going from structure-property prediction to generation and inverse design. This end-to-end framework demonstrates AI's role in automating both discovery and tuning of functional materials. Finally, Azher et al. [310] review the integration of ML in 4D printing, providing insights into how inverse design of time-evolving material behavior is becoming more systematic and data-driven.

AI-assisted design begins by training models on existing datasets, which may include both experimental results and simulation outputs. These datasets capture how different molecular motifs, processing parameters, or external stimuli influence material behavior such as deformation, stiffness modulation, or actuation speed. Foundational work by Sanchez-Lengeling and Aspuru-Guzik [311] remains pivotal, showing how generative models enable inverse molecular design, predicting functional molecules based on target property vectors with high generalizability across chemical spaces. Once trained, the AI can interpolate or even extrapolate these relationships to suggest new material designs likely to meet a given set of performance criteria. One of the most powerful capabilities of AI in this domain is its ability to navigate vast design spaces with high-dimensional input features, where human intuition or traditional modeling would struggle. Techniques such as generative adversarial networks (GANs), variational autoencoders (VAEs), and reinforcement learning are particularly well-suited for exploring novel architectures or stimuli-response pathways. In a comprehensive 2025 study, Shafiq et al. [312] developed a hybrid design framework using graph-based VAEs and GANs to generate stimuli-responsive materials directly from pixel-level representations. Their model successfully translates visual patterns into physical material architectures with target mechanical functions. Park et al. [313] evaluate whether generative AI has "solved" inverse materials design, reviewing how VAEs and GANs are reshaping discovery pipelines, especially in high-dimensional chemical and mechanical property spaces. Their review highlights how generative models outperform conventional sampling in proposing novel, valid, and synthesizable material structures. Würz and Weiβenfels [314] use deep reinforcement learning integrated with homogenization theory to design metamaterials with specific bulk responses. The agent iteratively learns design strategies based on performance feedback, mirroring biological evolution but with real-time convergence. Zeng et al. [300] propose a disentangled VAE for interpretable and data-efficient inverse design (Fig. 14(c)), enabling researchers to adjust latent variables directly linked to specific material behaviors such as stiffness or deformation rate. Long et al. [301] introduced ORGAN (Objective-Reinforced GAN), targeting inorganic solid materials (Fig. 14(d)). Their approach tailors GAN outputs to match predefined objectives like thermal stability or crystal lattice compatibility. These models can propose material compositions or structural geometries that achieve specified outputs under targeted mechanical or chemical inputs.

Importantly, AI does not function in isolation but is often coupled with physics-informed models, simulation pipelines, and experimental feedback loops. This hybrid approach sometimes called “physics-informed ML” ensures that the AI-generated predictions are physically meaningful and constrained by the laws of thermodynamics, mechanics, and chemical kinetics. A comprehensive review by Mogra and Jain et al. [315] highlights how hybrid AI-physics models are being applied to dynamic programmable systems, often by embedding physics-informed neural networks (PINNs) within finite element modeling pipelines. These hybrid models not only predict structural behavior under varying stimuli but also learn from experimental feedback to iteratively optimize system performance. They are especially well-suited for closed-loop control architectures in soft actuators and smart morphing surfaces. Such approaches enhance both accuracy and trustworthiness, especially when deployed in safety-critical applications like biomedical devices or soft robotics. A key practical limitation in the deployment of programmable materials through 4D printing lies in the inherent trade-off between fabrication speed, geometric precision, and material performance. Increasing printing speed often compromises spatial resolution and interfacial quality, which can adversely affect the accuracy of programmed shape transformations and actuation behavior. Conversely, achieving high precision typically requires slower processing and tightly controlled fabrication conditions, limiting scalability. In addition, material formulations that enable strong stimuli-responsiveness are not always compatible with rapid or high-resolution printing techniques. This “speed-precision-performance” trilemma is particularly critical in programmable materials, where the final functionality depends on both structural fidelity and time-dependent material response [316].

To address this challenge, recent efforts have focused on establishing process-structure-property-response closed-loop frameworks. In these approaches, printing parameters such as extrusion rate, curing intensity, and layer thickness are systematically linked to microstructural features, which in turn determine macroscopic mechanical properties and dynamic behavior. Machine learning plays a pivotal role by capturing complex, nonlinear relationships between processing conditions and functional outputs. By integrating experimental datasets, simulation results, and in situ monitoring data, ML models enable predictive optimization of printing parameters to achieve target responsive behaviors. Furthermore, emerging closed-loop control strategies incorporate real-time feedback during fabrication, allowing adaptive correction of deviations and improving reliability. These developments significantly enhance the practical applicability of programmable materials by enabling more scalable, precise, and performance-driven 4D printing processes. The integration of AI in programmable material design is still in its early stages, but the trajectory is clear. As datasets grow, modeling techniques mature, and interpretability improves, AI will become an indispensable tool not just for optimization but for creativity in materials science. The goal is to move toward “on-demand” materials that can be digitally specified and fabricated with minimal iteration, guided by intelligent design frameworks that learn from every experiment and simulation.

While AI-driven materials design offers unprecedented capabilities for inverse design and predictive optimization, several inherent limitations remain. Current models often require large, high-quality datasets for training, which are scarce for complex, multiscale, or chemically reactive systems. Generalizability outside the training domain can be limited, and interpretability remains a challenge, particularly for deep learning-based frameworks. Integrating AI predictions with experimental validation can be time-consuming, and hybrid physics-informed approaches are still in development [317]. Moving forward, advances in transfer learning, active learning, and self-improving generative models are expected to improve data efficiency, robustness, and predictive reliability. Coupling these developments with high-throughput experimentation and multiscale simulations will likely enable a new generation of AI-driven programmable materials design that is both accurate and forward-looking, guiding rational synthesis and functional deployment in diverse application domains.

To provide a clearer guideline for selecting appropriate modeling strategies, it is important to compare the capabilities and limitations of the different approaches discussed above. MD is particularly powerful for capturing bond activation, mechanophore response, and force-induced molecular transformations with high resolution. However, its high computational cost restricts simulations to relatively small systems and short timescales, limiting its direct applicability to macroscopic material behavior [318]. Coarse-grained models extend accessible length and time scales by reducing molecular detail, enabling simulation of larger systems, although at the expense of chemical specificity. In contrast, FEM are highly efficient for predicting macroscopic deformation, stress distribution, and structural performance under various loading conditions. Nevertheless, FEM inherently lacks chemical resolution and typically relies on phenomenological constitutive laws to represent chemomechanical coupling [319].

Coupled chemical-mechanical models partially bridge this gap by integrating reaction kinetics and transport phenomena with mechanical behavior, making them suitable for responsive gels and soft actuators, albeit with increased model complexity. Finally, machine learning approaches offer powerful tools for inverse design and rapid optimization across scales, but their performance depends strongly on data availability, model generalizability, and integration with physics-based constraints. Overall, these methods are complementary rather than competing, and effective modeling of programmable materials increasingly relies on multiscale and hybrid frameworks that combine their respective strengths.

## 7 Real-world applications

### 7.1 Soft robotics with chemically tunable stiffness

Soft robotics represents a transformative frontier in robotics, emphasizing flexibility, adaptability, and compliance with biological systems. A key enabler in this field is the development of materials with dynamically tunable stiffness, allowing robots to morph between soft and rigid states as tasks demand (Table 3). Chemical tuning via environmental stimuli such as pH, light, or redox potential offers a precise, localized method for altering mechanical properties in situ. Such capabilities expand the operational window of soft robots, enabling functions like delicate grasping or high-load support without the need for complex mechanical components [19,325,326].

**Table 3:** Representative real-world applications of chemomechanical materials across different domains.

Application Domain	Material Type	Mechanism of Actuation	Trigger Type	Functional Outcome	Ref.
Soft robotics	Ionic polymer-metal composites (IPMCs)	Ion migration and electroactive deformation	Electric field	Programmable motion, soft grippers	[320]
Biomedical implants	Shape-memory polymers	Thermal transition of polymer chains	Body temperature	Minimally invasive deployment	[321]
Chemomechanical sensors	Mechanophore-functionalized polymers	Force-induced color change or fluorescence	Mechanical stress	Damage sensing, strain visualization	[322]
Smart textiles	pH-responsive hydrogels	Swelling-induced textile stiffness modulation	Sweat pH variation	Responsive clothing, thermal comfort	[323]
Adaptive architecture	Magneto-responsive composites	Particle reorientation under magnetic field	Magnetic stimulus	Shape-changing façades, load adaptation	[324]

**Fig. 15:**
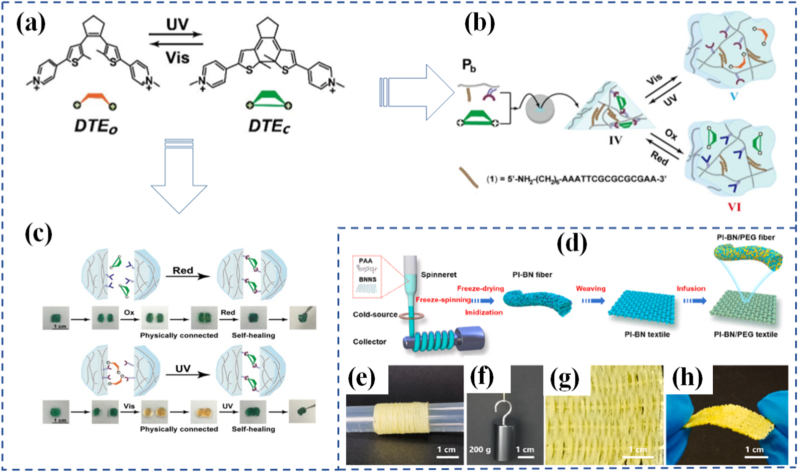
(a) Photoisomerization between DTEo and DTEc, (b) Schematic preparation and shape-memory properties of the hydrogel, (c) Self-healing of the dopamine/DTEc and self-complementary nucleic acid crosslinked hydrogel [328], (d) Schematic illustration of the preparation of PI-BN/PEG textile, (e) Digital photos of PI-BN aerogel fiber, (f) Optical image showing a PI-BN aerogel fiber hanging 200 g weight, indicating its high strength, and (g, h) Optical image of the as-prepared PI-BN/PEG textile [329].

Materials that exhibit stiffness modulation through chemical inputs often rely on reversible chemistries, such as dynamic covalent bonds or supramolecular interactions, to control cross-link density within a polymeric network. Hydrogels functionalized with ion-sensitive moieties can undergo stiffening or softening in response to electrolyte concentrations, a feature that mimics muscle-like actuation. For example, Tanaka et al. [327] demonstrated how stimuli-responsive hydrogels can replicate the dynamic stiffness of cellular microenvironments. Their hydrogels incorporate ion-sensitive and redox-reactive motifs, enabling reversible modulation of cross-link density in response to environmental cues. Li et al. [328] developed multi-stimuli-responsive hydrogels using a DNA/bipyridinium dithienylethene system (Fig. 15(a)). Their material responds to light, redox state, and chemical inputs, showing programmable shape-memory and self-healing behaviors via supramolecular cross-linking (Fig. 15(b and c)). Similarly, redox-active polymer networks have been developed where the oxidation state directly influences chain conformation and material modulus, providing a route for voltage-controlled mechanical transitions. Another highlight comes from Baretta and Frasconi [330], who engineered an electrically powered hydrogel with transient stiffness. Their work on redox-responsive disulfide cross-links allowed the material to function in out-of-equilibrium operations, which is critical for adaptive actuators and soft robotics. Finally, Xia et al. [331] presented a comprehensive review of macrocycle-based supramolecular polymer networks, describing how host-guest interactions and redox chemistry can be married with covalent backbones to achieve programmable, self-healing, and stiffness-tunable materials.

These chemically responsive systems are particularly advantageous in untethered or wearable robotics, where mechanical compliance is essential for safety and user comfort. Moreover, they enable feedback-driven motion, where chemical gradients in the environment or within the body can autonomously trigger shape changes or mechanical stiffening. For example, Yin et al. [332] review emerging systems where optical, microfluidic, and piezoresistive sensors are co-fabricated with soft robotic materials to provide real-time feedback and control. These embedded sensing mechanisms are essential in creating feedback-driven actuation in response to chemical gradients or ionic changes. In another example, Wang et al. [333] showcase PDMS-based microfluidic materials that tune stiffness dynamically via liquid metal channels, demonstrating promise for applications in healthcare and environmental sensing. Wang and co-workers presented a transparent and stretchable strain sensor utilizing single-walled carbon nanotubes (SWCNTs) as the conductive element. The SWCNT layer was transferred onto a PDMS substrate, forming a uniformly patterned sensing film that combined high optical transparency with excellent stretchability. Wang et al. [334] go further by integrating chemical feedback loops into wearable platforms, connecting biosensing capabilities with mechanical assistive functions, vital for real-time patient monitoring and therapeutic interventions. Recent advances also integrate such materials with embedded sensors and microfluidics, allowing the robotic system to respond intelligently to external stimuli in real-time. Materials reviewed by Dou et al. [326] also incorporate microfluidic channels to regulate internal stiffness and pressure, enabling shape change and grasping behaviors. These systems mimic biological muscles more closely and have immediate relevance in soft wearable robotic arms and exosuits. Overall, chemically tunable stiffness is reshaping how motion, sensing, and adaptability are engineered into soft robotic platforms. This approach circumvents the need for bulky actuators and provides new strategies for integrating control and structure into the same material body. Key performance metrics such as actuation strain, response time, and cyclic durability are critical in evaluating these systems, yet challenges remain in achieving fast response under low-energy input and maintaining long-term stability under repeated operation. As synthesis and control mechanisms become more refined, such chemomechanical materials are expected to become foundational components in next-generation robotic systems.

### 7.2 Mechanochemical sensors and actuators

Mechanochemical sensors and actuators harness the intrinsic coupling between mechanical force and chemical transformation, converting external stimuli such as stress or pressure into detectable chemical or electrical signals (Table 3). These systems often utilize force-responsive motifs like mechanophores, piezoelectric crystals, or stress-activated polymers that undergo bond rearrangement or charge separation under strain [[335], [336], [337]]. This mechanochemical transduction enables the creation of devices that can sense strain, fatigue, or damage and respond accordingly through visual cues, electronic outputs, or autonomous actuation. One compelling example includes materials embedded with spiropyran mechanophores that change color under mechanical load, offering real-time visualization of stress distribution or impending failure in structural components. This concept was deeply characterized in the study by Beiermann [338], which investigated mechanochemical activation in linear polymers and correlated optical response with localized stress fields, laying groundwork for force-reporting materials in engineering. In other designs, polymer composites exhibit changes in electrical conductivity or fluorescence as a function of deformation, allowing for integration with diagnostic electronics. Extending the concept into soft robotics and biohybrid systems, Li et al. [339] demonstrated a soft, robust mechanoluminescent material capable of dynamic mechanical sensing. Their system integrates mechanophores into a flexible network that responds to deformation with luminescent optical signals, offering potential for real-time damage mapping and signaling under biologically relevant conditions. These systems are especially promising in aerospace and civil engineering, where continuous health monitoring of materials can prevent catastrophic failure.

Actuation mechanisms based on mechanochemical principles can also deliver force on demand. For instance, stress-activated valves or pumps have been developed where a mechanical trigger initiates a chemical reaction that generates pressure, enabling fluid movement without electronics. A foundational contribution comes from the field of autonomic materials, as discussed in White and Sotos [340]. Their work introduces stress-activated valves and chemical release mechanisms that use stored chemical potential to initiate movement when triggered by mechanical deformation. This kind of force-on-demand architecture allows materials to perform autonomous mechanical work like pumping or clamping without requiring circuitry or external control. In microscale applications, this coupling becomes even more powerful, enabling bio-inspired motion and logic operations in constrained environments. For microscale systems, Huang et al. [341] review bioinspired chemical actuation across length scales, including mechanisms where stress or pressure gradients trigger chemical changes, leading to localized swelling, fluid flow, or shape change. These systems are critical for microfluidic transport, chemical logic gates, and autonomous motion in confined environments where traditional actuation (motors, hydraulics) is unfeasible. The growing convergence of materials science, chemistry, and electronics has led to highly multifunctional systems, where sensing, computation, and response are embedded in a single material layer. As sensor resolution and actuation precision improve, mechanochemical devices are expected to play a central role in future autonomous systems, wearables, and responsive environments.

### 7.3 Smart textiles and adaptive architecture

Smart textiles and adaptive architectural elements represent a rapidly growing application area for programmable materials, especially those with chemomechanical functionality (Table 3). These systems aim to create environments and garments that can adjust their form or function in response to external conditions, including temperature, humidity, light, or pollution levels. At the heart of these systems are fabrics and structures embedded with responsive materials that allow for dynamic ventilation, insulation, or structural transformation [[342], [343], [344]].

In textiles, fibers containing shape-memory polymers or phase-change materials enable garments that adjust their porosity or stiffness in response to body temperature or environmental changes. This feature is particularly relevant for performance wear and medical garments, where dynamic comfort and support are needed. In a comprehensive review, Ornaghi and Bianchi [345] explain how electrospinning, wet spinning, and melt spinning techniques are being optimized to incorporate SMPs into fibers. These materials exhibit reversible stiffness modulation and shape recovery, allowing garments to deliver zonal support or breathability in response to temperature changes. Their work also addresses biomedical integration, highlighting the potential for responsive bandages and compression wear. Peng and Cui [346] discuss thermal management in nanofiber-based fabrics, combining phase-change materials (PCMs) and (SMPs) to enable clothing that regulates heat transport and conversion. These fibers can be manufactured using electrostatic spinning, creating ultrafine filaments that adjust fabric porosity or thermal insulation on demand. Advances in fiber spinning and 3D knitting have made it possible to distribute responsive materials at microscale resolutions, allowing for complex, zonal control over fabric properties. Zhang [347] demonstrates how 3D knitting techniques are being paired with thermoresponsive gels and phase-change materials to produce adaptive soft robotic fabrics. This approach enables zonal thermal regulation and mechanical response distributed across fabric surfaces ideal for athletic or therapeutic use. In related work, Tang [348] highlights the integration of aerogel-infused fibers and wet-spun PCMs, creating textiles with enhanced thermal buffering. Using a freeze-spinning approach, researchers fabricated polyimide-boron nitride (PI-BN) composite aerogel fibers that combine high porosity with excellent mechanical strength. These fibers serve both as an efficient thermal conduction network and as a robust scaffold for incorporating PCMs. To prepare a phase-change-active textile, polyethylene glycol (PEG) was infused into the porous PI-BN aerogel fabric through vacuum-assisted impregnation (Fig. 15(d–h)). The interconnected PI-BN structure, with its superior thermal conductivity, enables fast heat transfer during phase transitions, while its porous architecture accommodates a high PEG content and prevents leakage of the molten phase. The resulting PI-BN/PEG composite fabric demonstrated a thermal conductivity of 5.34 W m^−1^ K^−1^, a high latent heat of 125.2 J g^−1^, and maintained stable performance over 100 thermal cycling tests. Furthermore, applying a thin polydimethylsiloxane (PDMS) layer to the surface rendered the textile water-repellent and washable [329]. This design enables dynamic response to fluctuating external temperatures while maintaining breathability and flexibility.

**Fig. 16:**
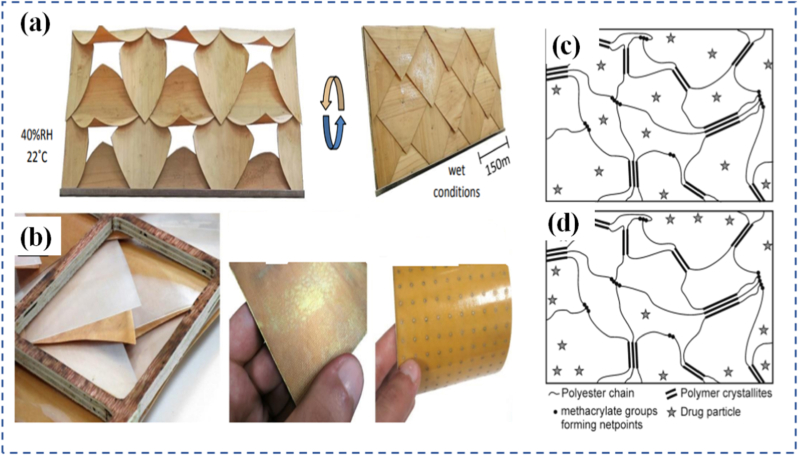
(a) An early prototype of a responsive cladding module, which incorporates panels with glued plain-cut European maple (*Acer pseudoplatanus*) inlay veneer active layers and 0.5 mm thick polycarbonate passive layers, shown in dry (left) and wet states (right), (b) Severe delamination of the layers of prototype 1 panels, glued with one of the initially selected adhesives [350], (c) Scheme on homogeneous distribution or surface-near localization, and (d) Drug particles (amorphous aggregates or crystals) in a cross-sectional view of polymer network films [351].

Adaptive architecture extends these principles to building materials and structural components. Panels incorporating humidity-sensitive hydrogels can open or close ventilation flaps autonomously, regulating indoor air quality and energy use. One prominent example is the development of hydrogel-based smart windows, as described by Zhou et al. [349], where humidity-responsive interpenetrating networks (IPNs) modulate transparency and light transmission in response to environmental moisture levels. These systems provide passive climate control by adjusting thermal and optical properties without requiring electronic input. In a broader architectural context, Holstov [350] explored wood-based hygromorphic materials that bend or unfold with humidity changes (Fig. 16(a and b)). These panels can act as breathing skins or autonomous ventilation flaps, dynamically controlling airflow to reduce energy consumption while improving comfort and air quality. Similarly, surfaces that change transparency or color with temperature can improve thermal efficiency and aesthetics without external power sources. Thermochromic surfaces also offer promise for smart thermal management, as detailed in An et al. [352]. Their work examines switchable radiative coatings that alter their emissivity and color based on temperature, effectively reducing solar heat gain during hot periods and improving insulation when it's cold entirely without external power sources. Similarly, Shen and Li [353] reviewed liquid crystal-based smart windows capable of passive light modulation via thermally driven phase transitions, combining energy efficiency with aesthetic adaptability. These innovations are driven by the increasing need for sustainable, low-energy solutions in urban design. The interdisciplinary nature of smart textiles and adaptive architecture blending design, material science, and environmental engineering demands novel approaches to material synthesis and integration. As these systems evolve, future cities and living spaces may become more responsive, autonomous, and interactive, shaped by the ongoing fusion of chemistry and mechanics.

### 7.4 Shape-memory materials in biomedical devices

Shape-memory materials (SMMs) are a class of smart materials capable of undergoing programmed deformation and returning to their original shape upon stimulation. In biomedical applications, SMPs and alloys (SMAs) are widely used to deploy devices within the body in a minimally invasive form and then trigger a conformational change via temperature, light, or chemical environment. This transformative behavior is particularly valuable in applications such as stents, sutures, and scaffolds, where precision, biocompatibility, and responsiveness are vital. A foundational review by Zhao et al. [354] provides a comprehensive overview of SMPs and their composites in medical applications, particularly focusing on tissue engineering scaffolds, biological sutures, and stents. These materials undergo a predefined shape transformation upon exposure to physiological stimuli, allowing for device insertion in a compact form and subsequent expansion or conformational change once deployed. In more targeted use, Yakacki and Gall [355] demonstrated SMPs for sutures and vascular stents that respond to body heat, thus offering automated closure or support without the need for manual manipulation. Similarly, Chen et al. [356] developed a temperature-responsive biodegradable SMP suture capable of closing wounds post-activation, simplifying surgical procedures. Moving toward next-gen applications, Delaey and Dubruel [357] report on 3D-printed SMP scaffolds with magnetic responsiveness, enabling remote activation via external fields. These systems can support minimally invasive interventions while allowing real-time control over scaffold geometry and stiffness. Wu et al. [358] provide a roadmap for using SMPs and SMAs in bone repair, vascular implants, and nerve regeneration, emphasizing that temperature, pH, or light can all serve as programmable triggers for activation. Their work highlights the role of stimuli-specific scaffolds in complex healing environments.

Chemically responsive SMPs are designed to activate based on physiological stimuli like pH or ion concentration, making them particularly suited for site-specific therapeutic interventions. Singhwane et al. [359] provide a detailed review of smart SMPs tailored for activation via pH changes in wound microenvironments. These systems are ideal for self-tightening sutures that contract in acidic conditions, promoting automated wound closure without external input. Similarly, thermally activated SMP stents can be inserted in a collapsed form and expand at body temperature to restore vessel patency. Sun et al. [360] highlight how biodegradable SMP stents can be activated by physiologically relevant triggers like temperature and ion concentration. These materials allow insertion in a collapsed state and then expand autonomously at body temperature to restore blood vessel patency. Pisani et al. [361] demonstrate nanofiber-based SMPs for vascular devices and internal suturing, which are activated by chemical stimuli such as pH or ionic gradients. These systems integrate antimicrobial properties, further enhancing their biomedical utility.

Designing such systems requires a deep understanding of both material chemistry and the mechanics of deformation and recovery. A key challenge in these systems is the inherent trade-off between reversibility and long-term mechanical stability. While dynamic covalent bonds and supramolecular interactions enable self-healing and reprogrammability, their reversible nature can lead to reduced mechanical strength, creep, or fatigue over extended use. Recent research has focused on addressing this bottleneck through molecular design strategies, such as incorporating dual-network architectures that combine permanent covalent cross-links with reversible dynamic bonds, thereby balancing structural integrity with adaptability. In addition, tuning cross-linking density and introducing hierarchical bonding motifs can improve load-bearing capacity while preserving reversible functionality [262]. External energy input strategies, including light, thermal, or electrochemical activation, have also been employed to selectively trigger bond exchange or healing processes only when needed, minimizing unintended relaxation under service conditions. These approaches collectively aim to decouple reversibility from mechanical degradation, enabling programmable materials that are both durable and reconfigurable. The molecular architecture, such as the inclusion of soft segments and reversible cross-links, dictates the material's shape-memory behavior and recovery speed. Moreover, the integration of drug delivery functionalities into these materials has broadened their application scope. Wischke et al. [351] further illustrate how drug incorporation techniques, such as blending before cross-linking or post-synthesis swelling, influence both release kinetics and shape-memory recovery speed (Fig. 16(c and d)). Embedding soft segments like polycaprolactone increases elasticity and improves drug dispersion, while rigid segments define the recovery profile. Additionally, Peterson and Dobrynin [362] developed biodegradable SMPs with dual functionality: their mechanical expansion under physiological temperature aligns with controlled degradation, creating a time-programmed drug-release profile alongside structural support. From orthopedic implants to tissue engineering scaffolds, the adaptability of shape-memory materials is redefining how mechanical support and biological interaction are co-engineered. An emerging extension of these concepts is found in 4D tissue implants, where cell-laden hydrogels are engineered to undergo programmed morphological and functional evolution over time. In such systems, biological processes such as chondrogenesis and osteogenesis are integrated with stimuli-responsive material behavior, enabling the formation of cartilage and bone constructs with dynamically evolving architectures. These 4D constructs demonstrate how programmable materials can guide tissue maturation, structural organization, and functional integration within physiological environments. Incorporating this approach further highlights the potential of programmable materials in advanced regenerative medicine and next-generation biomedical implants. As fabrication techniques such as 4D printing evolve, the ability to design and deploy personalized biomedical devices with tailored responsiveness is rapidly becoming a reality. Despite these advances, several limitations remain, including the trade-off between biocompatibility and mechanical strength, limited long-term stability in physiological environments, and challenges in precise spatiotemporal control of activation.

## 8 Machine intelligence in metamaterials design

The emergence of machine intelligence in metamaterials marks a fundamental shift in how programmable matter is conceived, designed, and deployed. This shift is not unidirectional: while AI accelerates the discovery and optimization of metamaterials, metamaterials themselves are increasingly recognized as platforms for computation and intelligence, particularly in the realm of mechanical computing and robotics. This dual framework AI for metamaterials and metamaterials as AI highlights the transformative synergy between data-driven design and material-embedded logic.

Beyond structural and geometrical optimization, recent advances highlight the growing role of artificial intelligence in mechanochemistry at the molecular scale. Machine learning approaches are increasingly being applied to accelerate the discovery of mechanophores by screening large chemical spaces and identifying molecular motifs that exhibit force-responsive behavior [363]. By learning structure-property relationships from both experimental and computational datasets, these models can predict activation forces, reaction thresholds, and functional outputs, significantly reducing the reliance on trial-and-error synthesis [364].

AI has also shown strong potential in predicting reaction pathways under mechanical force. Traditional quantum chemical methods for mapping force-modified energy landscapes are computationally demanding, particularly for complex systems. In contrast, machine learning potentials and surrogate models can approximate these energy surfaces with significantly reduced computational cost, enabling rapid exploration of force-induced reaction coordinates and mechanochemical pathways. Furthermore, hybrid frameworks that integrate physics-based modeling with machine learning are emerging as powerful tools for capturing force–chemical coupling across scales. For instance, physics-informed neural networks (PINNs) and data-driven constitutive models can incorporate mechanochemical kinetics into continuum simulations, allowing for more accurate prediction of stress-dependent reaction rates and material response [365]. These approaches are particularly relevant for designing adaptive polymers and hydrogels where chemical transformations directly influence mechanical behavior. Together, these developments demonstrate that AI is not only transforming structural design but also enabling a deeper understanding and control of mechanochemical phenomena, paving the way toward truly programmable materials at the molecular level [365].

### 8.1 AI for intelligent metamaterials

AI-driven strategies have dramatically expanded the design space of programmable metamaterials. ML and physics-informed approaches can predict mechanical, optical, and chemical responses from structural descriptors, while inverse design workflows enable researchers to specify a target function (e.g., tunable stiffness, memory effects, or nonlinear acoustic behavior) and let algorithms generate candidate architectures. Yan et al. [366] developed a deep learning framework for the inverse design of acoustic metamaterials with highly customizable absorption spectra. Their model rapidly identified structural architectures corresponding to desired absorption peaks, effectively bypassing the need for computationally expensive simulations. This approach greatly enhanced the exploration of high-dimensional design spaces and offered practical strategies for noise control and soundproofing applications. By leveraging prior acoustic knowledge, the authors constructed a specialized dataset that incorporated both closed rigid and thin-walled inclusions to achieve broadband, low-frequency sound absorption. Using a conditional generative adversarial network (cGAN), the model learned the nonlinear relationship between target broadband absorption spectra and metaporous materials with perforated plate configurations. The inverse-designed structures demonstrated high accuracy, achieving a mean squared error (MSE) of 5.03 × 10^−3^ and a mean absolute percentage error (MAPE) of 6.39% between the predicted and target absorption spectra. Such workflows overcome the limitations of brute-force simulations and trial-and-error experiments. Farajollahi et al. [367] combined deep neural networks with particle swarm optimization to design acoustic metamaterials featuring tailored bandgaps. Their integrated framework effectively predicted structure property relationships and generated novel unit cell geometries with targeted acoustic filtering capabilities, demonstrating significantly higher efficiency compared to brute-force search methods. To address design challenges more efficiently, the authors applied various machine learning algorithms including random forests, extra trees, k-nearest neighbors, and artificial neural networks to predict dispersion bandgaps in cylindrically pillared acoustic metamaterials. The study considered three primary design parameters: the ratios of substrate layer thickness, cylinder diameter, and cylinder height to the unit cell length. After comprehensive hyperparameter tuning and model training, the best-performing model was achieved using a deep learning architecture (multi-layer artificial neural network), which attained a high determination coefficient (R^2^ = 0.997). Furthermore, the trained models were successfully employed for the inverse design of cylindrically pillared phononic crystals targeting four distinct bandgap ratios. The developed artificial neural network exhibited the highest predictive accuracy, achieving an R^2^ of 0.998. Zhang et al. employed a generative adversarial framework consisting of a generator-simulator-critic architecture for the inverse design of acoustic metamaterials. This system efficiently produced unit cell structures with tailored performance metrics, outperforming conventional optimization methods. The results demonstrated excellent agreement between the designed and target frequency responses, underscoring the potential of AI-driven generative models in practical acoustic device design. Specifically, the authors investigated the capability of their proposed inverse design framework to enhance the acoustic performance of a three-dimensional mixed-size cavity-based waterborne sound absorptive metamaterial. Within this framework, the target sound absorption spectra (100-10,000 Hz) were provided as inputs to the inverse network, which generated corresponding structural designs exhibiting optimal spectral matches. These structures were then evaluated by the forward network to assess acoustic properties and compute loss functions. The proposed approach achieved a remarkable improvement in inverse design accuracy, increasing the proportion of cases with mean squared errors below 0.0001 from 9.2% to 99.6%, while also enabling beyond-range design exploration [368].

High-throughput screening, combined with reinforcement learning, has already been applied to design metamaterials with programmable memory and malleability, where structural reconfigurations can be encoded, erased, and reprogrammed. PINNs further ensure that predictions respect governing mechanochemical laws, enhancing the reliability of AI-guided design. Kang et al. [369] applied PINNs to model and inversely design origami-inspired metamaterials featuring reprogrammable folding patterns. Their approach incorporated essential mechanical constraints such as kinematic compatibility and elastic energy conservation directly into the training process, ensuring physically consistent predictions. Simulation results demonstrated over 95% accuracy in predicting folding trajectories, while the inverse-designed structures successfully achieved targeted shape transformations under external loading conditions. This framework effectively enables memory-like encoding within origami lattice architectures, paving the way for mechanically intelligent and reconfigurable metamaterials. Zhang et al. [370] combined physics-informed machine learning with topology optimization to design reconfigurable metasurfaces for IoT communications, demonstrating both efficiency and scalability. Their PINN-based framework reduced design time by approximately 80% compared to conventional brute-force electromagnetic solvers, while numerical simulations showed around a 15% improvement in beamforming efficiency and maintained consistent physical feasibility across frequency bands, ensuring robust reconfigurability with strict compliance to Maxwell's equations. Furthermore, they demonstrated inverse design of metasurfaces capable of multi-bit programmability (0/1/2 states). The PINN-based optimizer lowered computational costs by roughly 90% compared to traditional FDTD sweeps. The designed metasurfaces exhibited over 98% accuracy in beam-steering tasks, which was experimentally verified in microwave regimes, providing a scalable approach for adaptive wireless communication and optical memory functions [371].

### 8.2 Generative and hybrid design strategies

GANs, VAEs, and evolutionary algorithms are particularly effective in exploring unconventional architectures. These approaches have revealed designs for metamaterials with negative Poisson's ratio, multistable states, or stress-triggered color change that defy conventional intuition. When coupled with finite element simulations, such models form hybrid AI-mechanics frameworks capable of bridging molecular-level chemistry with macroscale mechanics. Li et al. [372] employed a VAE to generate absorber unit-cell designs, which were subsequently optimized using FEM simulations. Their framework achieved absorptivity greater than 95% within the 8-12 GHz frequency band, producing structural configurations that had not been previously reported. By reducing the design search space by approximately 70%, the VAE significantly accelerated the optimization process, demonstrating the effectiveness of generative modeling for broadband absorber design [373]. Wang et al. [374] developed a Gaussian-mixture β-variational autoencoder (β-VAE) to explore and inversely design novel phononic crystal structures. Their framework successfully generated unit cell geometries with tunable bandgaps, achieving bandgap widths of up to 32% of the central frequency as validated by FEM simulations. The incorporation of iterative model updating ensured stable convergence, even under sparse training conditions. In a related study, On et al. [375] proposed a VAE-based latent search strategy for designing pixelated microwave absorbers. The generated absorber patterns were optimized for maximum absorption within X-band frequencies, achieving absorptivity greater than 90% across a 4 GHz bandwidth. This approach outperformed traditional random search methods, reducing computational costs by approximately 60% while maintaining high design accuracy and broadband performance.

**Fig. 17:**
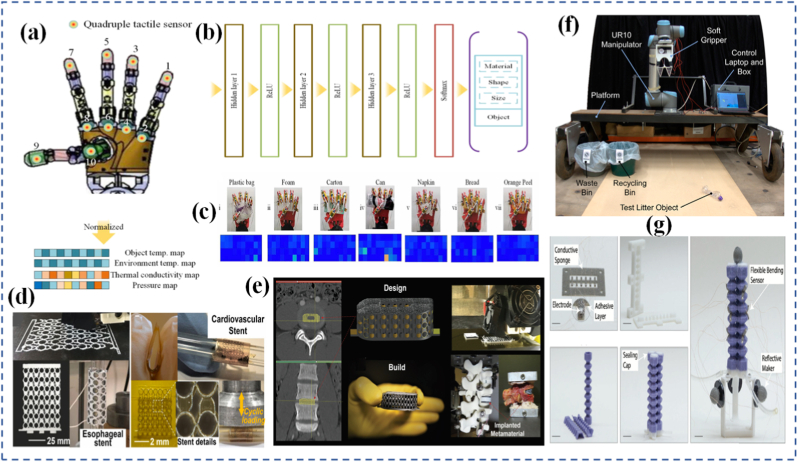
(a) Schematic diagram of the robot hand integrated with 10 quadruple tactile sensors, (b) Schematic diagram of the multilayer perceptron model structure for identifying object material, shape, and size, (c) A group of example signal maps when the robot hand grips seven types of garbage [376], (d) Design details and testing of the cardiovascular stent film [377], (e) Fabrication process of the proposed self-aware metamaterial implants [378], (f) The LitterBot and its components [379], and (g) Fabrication of flexible bending sensor and pneumatic soft actuator [380].

For mechanochemical systems, AI-guided generative design can optimize the incorporation of mechanophores, supramolecular motifs, or polymer blends to achieve tunable elasticity, self-healing, or controlled degradation. This enables inverse design workflows where chemomechanical responsiveness is directly encoded into lattice architectures. In this context, Zhu et al. [376] developed a robotic hand equipped with quadruple tactile sensors capable of accurately recognizing objects during grasping. Initially, the authors identified the most frequently contacted areas of the robotic hand when grasping various objects. Based on this analysis, ten quadruple tactile sensors were strategically installed on the five fingertips and palm of the humanoid robotic hand, as shown in Fig. 17(a). The four-row signal graph from top to bottom represents the sensor outputs corresponding to object temperature, ambient temperature, object thermal conductivity, and contact pressure. To train the robotic hand for object recognition, experiments were conducted at room temperature, during which the hand grasped a human hand and twelve other standard objects differing in size (small and large), shape (cube and sphere), and material (steel, acrylonitrile butadiene styrene, and sponge). The corresponding tactile sensor signals were recorded for each case. Within the PyTorch framework illustrated in Fig. 17(b), a multilayer perceptron (MLP) model with three hidden layers was employed to classify objects based on their size, shape, and material, achieving an overall classification accuracy of approximately 96%. Moreover, the intelligent robotic hand was further utilized for a garbage classification task (Fig. 17(c)), where it demonstrated a recognition accuracy of 94% across seven types of waste materials.

### 8.3 Metamaterials as computing platforms

Beyond serving as objects of design, metamaterials themselves can act as computing substrates. Recent advances in mechanical computing have shown that carefully architected lattices, beams, and kirigami/origami systems can perform Boolean logic, memory storage, and signal processing directly through mechanical interactions [381]. Programmable metamaterials have been demonstrated as mechanical logic gates, multistable switches, and even analog computing units, offering robust and energy-efficient alternatives to conventional silicon-based systems. Zou et al. [382] introduced modular mechanical computing units capable of performing multi-input logic and memory operations through programmable metamaterial lattices. Each logic gate functions entirely via elastic deformation, exhibiting measured switching thresholds in the range of 0.8-1.2 N. A coupled network of these units demonstrated real-time signal propagation with response times below 100 ms. The proposed architecture enables cascaded logic operations and reprogrammable circuit configurations, offering a foundation for adaptive and mechanically driven computation. Chen et al. [383]introduced mechanical transistors that integrate binary switching and memory retention within a single metamaterial element. The system exhibited stable bistability with retention times exceeding 10^5^ cycles and demonstrated negligible mechanical fatigue. Fundamental logic operations, including NAND and NOR, were achieved through strain propagation between adjacent cells. This innovation bridges the gap between computation and memory, paving the way for fully mechanical processors capable of operating in harsh or electronics-restricted environments. Furthermore, as illustrated in Fig. 17(d and e)), a new generation of multifunctional metamaterial-based implantable devices has been developed, capable of autonomously responding to environmental changes and monitoring internal conditions. Emerging studies in this domain highlight how miniaturized cardiovascular stents (Fig. 17(d)) can be designed to track arterial radial pressure variations caused by tissue overgrowth without the need for external electronics [377], while self-powered mechanical metamaterial spinal fusion constructs (Fig. 17(e)) enable real-time monitoring of healing progress [378]. Mei et al. [384] engineered a reprogrammable metamaterial capable of performing digital logic operations such as AND, OR, and XOR through controlled buckling states. The unit cells encoded binary information via geometric bistability, while logic reconfiguration was achieved by modulating local strain fields, enabling real-time programming of computational logic. Experimental evaluations confirmed logical accuracies exceeding 95% across 500 operational cycles, demonstrating robust and repeatable mechanical computation. In a subsequent seminal study, Yasuda et al. [385] realized fully mechanical computing circuits integrating logic gates, memory, and sequential logic within architected metamaterial systems. Memory bits (m-bits) were implemented using multistable structural modules, facilitating complex operations such as addition and counting. The mechanical computers processed binary input sequences with switching times below 0.2 s, achieving fault-tolerant, all-mechanical computation entirely without electronic components.

In robotics, programmable responsive metamaterials enable actuators, grippers, and locomotion systems that not only respond to stimuli but also process mechanical information. Such systems blur the boundary between structure and computation: the metamaterial is simultaneously the sensing element, the logic processor, and the actuator. This concept, often described as “embodied intelligence,” eliminates the need for external controllers, paving the way for robots that think through their material architecture rather than electronic circuits. Lin et al. [386] reported a robotic skin constructed from programmable haptic metamaterials that integrate distributed sensing, mechanical logic, and adaptive feedback. Utilizing an AI-augmented mechanical feedback loop, the material autonomously decoded tactile stimuli with an accuracy exceeding 94%. The system exhibited rapid, millisecond-scale responsiveness (<20 ms), enabling real-time adaptation during grasping tasks. This design demonstrates the full embodiment of perception, computation, and response within a single material network, advancing the concept of intelligent matter for soft robotics. In a related study, Wu et al. [387] introduced a vision-guided metamaterial gripper that unifies tactile sensing, mechanical computation, and actuation. The gripper analyzed deformation patterns optically to achieve autonomous grasp classification and self-adjustment without relying on electronic sensors. Experimental tests on diverse objects revealed a 98% grasp success rate and adaptive stiffness modulation ranging from 20 to 120 kPa. This work underscores the integration of embodied vision and mechanical intelligence as a pathway toward fully autonomous and adaptive soft robotic manipulation.

### 8.4 Toward adaptive and autonomous metamaterials

The integration of AI-driven design and mechanical computing capabilities sets the stage for adaptive and autonomous metamaterials. Digital twins virtual counterparts of physical metamaterials can continuously update predictions based on experimental data, enabling real-time reconfiguration under external stimuli [388]. Reinforcement learning frameworks have already been applied to soft robotic metamaterials, allowing them to refine actuation strategies autonomously [389]. Almanzor et al. [379] developed a robotic system named LitterBot, designed for autonomous garbage collection. The robot is equipped with a fin-ray-type soft gripper integrated with a camera (Fig. 17(f)). Its machine vision system employs the Detectron2 implementation of the Mask Region-Based Convolutional Neural Network (Mask R-CNN) to classify and segment ground objects, as well as to determine their angular orientation for precise grasping. The convolutional neural network was trained using the Trash Annotations in Context (TACO) dataset, which contains images of waste objects captured by the robot's integrated camera. Furthermore, Zhang et al. [324] investigated a hybrid robotic system comprising electrically actuated robotic platforms and pneumatically actuated soft manipulators. The study evaluated the effectiveness of various sensor-derived inputs for neural network control and proposed several models-most notably a long short-term memory (LSTM) network-to achieve accurate positioning and control of soft robotic systems. Shu et al. [380] employed a long short-term memory (LSTM) network to establish the correlation between signals obtained from flexible porous piezoresistive sensors and the positional state of a soft actuator (Fig. 17(g)). The network inputs consisted of data from individual sensors made of a thin conductive sponge material capable of detecting bending and positional changes, while the output represented the actuator's absolute spatial position. Through this approach, the LSTM network effectively modeled and predicted the positional behavior of the soft actuation system. At the same time, metamaterials with built-in computing ability can serve as distributed decision-making systems, processing sensory inputs and triggering programmed responses without centralized electronics. Coupled with mechanochemistry, such systems could evolve into self-regulating, self-learning materials capable of sustainable operation in dynamic environments [390].

## 9 Scalability and sustainability of programmable materials

Despite rapid advances in programmable materials, the transition from laboratory-scale demonstrations to real-world deployment remains a major challenge, particularly in terms of scalability and sustainability. From a manufacturing perspective, many programmable systems rely on complex synthesis routes, multi-step functionalization, or precise spatial patterning (e.g., in mechanophore incorporation or 4D printing architectures), which are difficult to reproduce at industrial scale. Ensuring uniform force transmission pathways, consistent cross-linking density, and defect-free architectures across large volumes remains a key bottleneck [391,392].

Scalability challenges are further exacerbated in systems that integrate multiple functionalities such as sensing, actuation, and self-healing within a single material platform. These systems often require hierarchical structuring across molecular, micro-, and macro-scales, making fabrication both time- and resource-intensive. Emerging strategies such as modular synthesis, additive manufacturing, and roll-to-roll processing are being explored to address these limitations, but their compatibility with complex mechanochemical functionalities remains an open question.

From a sustainability perspective, the environmental footprint of programmable materials is an increasing concern. Many current systems rely on energy-intensive fabrication processes, non-renewable precursors, or chemically irreversible components that hinder recyclability. In particular, materials based on permanent covalent networks may offer excellent mechanical robustness but pose challenges for reprocessing and end-of-life management. To address this, recent research has focused on incorporating dynamic covalent bonds or supramolecular interactions that enable reconfigurability, self-healing, and recyclability [393]. However, these approaches often introduce a trade-off between reversibility and long-term mechanical stability.

Another important direction involves the development of closed-loop material systems, where degradation, reconfiguration, or recycling can occur with minimal environmental impact. This includes the use of bio-derived polymers, green synthesis pathways, and stimuli-responsive systems that operate under low-energy inputs. Furthermore, integrating life-cycle assessment (LCA) into the design process is becoming increasingly important for evaluating the true sustainability of programmable materials [394]. Overall, addressing scalability and sustainability requires a holistic approach that combines advances in chemistry, processing technologies, and systems-level design. Bridging these aspects will be essential for translating programmable materials from proof-of-concept systems to practical, deployable technologies.

### 9.1 Challenges and future directions

Despite the remarkable progress in the field of programmable materials, several challenges continue to limit their widespread deployment. One of the foremost issues is scalability. Many programmable systems, especially those involving intricate mechanochemical or stimuli-responsive architectures, are demonstrated at the laboratory scale with carefully controlled conditions. Translating these principles into industrial-scale production remains non-trivial, primarily due to the complexity of integrating responsive components without compromising material robustness or function. Additionally, repeatability is often a concern, particularly when molecular-level triggers are employed. Variability in environmental conditions, processing parameters, or the mechanical history of a material can lead to inconsistencies in performance. Ensuring that programmable responses are reliable over multiple cycles and in diverse settings is critical for real-world applications, especially in safety-critical domains like biomedical implants or aerospace actuators.

Environmental and energy considerations present another layer of complexity. The fabrication of some programmable materials involves high energy input, toxic precursors, or rare-earth components, all of which raise sustainability concerns. Furthermore, many actuation mechanisms still rely on external power sources, such as electrical fields, thermal gradients, or chemical reagents that are not always sustainable or recyclable. As the field moves forward, there is a growing need to develop materials and systems that are both energy-efficient and environmentally benign. This includes not only the use of green synthesis methods but also the design of closed-loop systems where materials can degrade harmlessly or be reconfigured without external waste or inputs.

A major frontier in the evolution of programmable materials is the integration of digital logic with matter itself. This involves embedding computational capabilities directly into materials, enabling them to sense, process, and respond to environmental stimuli autonomously. While some progress has been made using embedded electronics or soft logic circuits, the full realization of digital-material convergence remains aspirational. Challenges lie in the miniaturization, energy autonomy, and biocompatibility of such integrated systems. Moreover, embedding computation within materials requires a reconceptualization of both hardware design and material composition, creating the need for interdisciplinary research at the interface of material science, electronics, and informatics.

Here, machine intelligence offers both opportunities and challenges. On one hand, data-driven approaches can accelerate the discovery of new mechanochemical motifs, optimize architectures for multifunctional performance, and guide inverse design strategies that would be intractable with conventional trial-and-error methods. On the other hand, several critical limitations remain. One major challenge is the limited availability of high-quality, standardized datasets for programmable materials, particularly for systems involving coupled chemical–mechanical behaviors across multiple scales. In addition, model generalizability remains a concern, as many machine learning models trained on specific material classes or conditions may not readily transfer to new systems or environments. Another key issue is the gap between computational predictions and experimental validation, where discrepancies can arise due to simplifications in modeling or uncertainties in material synthesis and characterization. Addressing these challenges will require the development of robust databases, standardized descriptors, and hybrid frameworks that integrate physics-based modeling with machine learning, ensuring that AI-driven approaches provide reliable and experimentally actionable insights.

Perhaps the most visionary and transformative direction lies in the development of autonomous, self-regulating materials. These are materials that can not only respond to stimuli but also adapt, heal, and evolve in response to changing conditions. Inspired by biological systems, such materials would possess feedback loops, homeostatic mechanisms, and decision-making protocols at the molecular or microscale. Realizing this vision involves deep integration of sensing, actuation, logic, and memory all within the material itself. It will require the convergence of synthetic biology, AI, and soft robotics to create living-like materials capable of sustainable operation in dynamic environments.

Looking ahead, programmable materials hold the potential to redefine how we design and interact with matter. From soft robots that adapt their stiffness in real-time, to building materials that adjust their insulation properties based on weather, to wearable devices that sense and respond to biochemical signals, the possibilities are vast. However, unlocking these capabilities requires not only technological advances but also new frameworks of collaboration, regulation, and education. By addressing current challenges head-on and embracing an interdisciplinary ethos one that fully integrates mechanics, chemistry, and machine intelligence the next generation of programmable materials could usher in a new paradigm where matter is not just passive but participatory in the systems it inhabits.

## 10 Conclusion

This review has explored the intricate and multifaceted world of programmable materials, emphasizing the underlying mechanisms that allow them to respond intelligently to external stimuli. We examined how chemical signals, mechanical deformation, and molecular architectures converge to produce dynamic, adaptable behavior in soft matter systems. From covalent and supramolecular chemistries that drive force generation to strain-tunable microstructures and digital modeling techniques, the various components do not operate in isolation. Instead, they form synergistic frameworks where the mechanical and chemical domains reinforce and inform each other. These relationships, grounded in fundamental molecular interactions, give rise to material systems capable of performing complex tasks such as actuation, sensing, and self-regulation.

Looking forward, the most promising pathways for industrial translation lie in mechanochemical systems that combine robustness, scalability, and predictable coupling between force and chemical reactivity. In particular, stress-activated covalent chemistries (mechanophores), reversible supramolecular networks, and mechanically gated polymer architectures stand out as leading candidates for real-world deployment. Mechanophore-based polymers offer direct force-to-chemical signal conversion, enabling damage sensing, self-reporting, and adaptive response under operational loads. Similarly, supramolecular systems based on hydrogen bonding, metal-ligand coordination, and host-guest interactions provide reversible and energy-dissipating mechanisms that are highly attractive for recyclable elastomers and adaptive coatings. In parallel, strain-engineered microstructured materials and fiber-reinforced composites provide scalable routes for integrating mechanochemical functionality into load-bearing components.

The future of programmable materials lies in advancing this synergy even further. As researchers refine the design principles of force-responsive polymers, develop more accurate multiscale simulations, and integrate machine intelligence into materials discovery and optimization, we are moving toward a new generation of intelligent matter. Data-driven design strategies, generative algorithms, and digital twins provide powerful tools to accelerate discovery, predict performance, and enable adaptive, self-optimizing systems. These next-generation materials will not only respond passively to cues but will actively participate in decision-making processes, adapting their structure, function, or even chemistry in response to real-time feedback. This capability opens doors to transformative technologies in biomedicine, soft robotics, aerospace, and smart infrastructure, where materials can autonomously adjust their properties to suit evolving conditions.

A key vision emerging from this work is the integration of programmable behavior across molecular, mesoscopic, and macroscopic scales within unified material systems. Such materials would transcend current static limitations, exhibiting life-like properties such as memory, adaptability, and resilience. Achieving this will require new chemistries, responsive networks, computational frameworks, and control architectures embedded within the material's structure. Bridging digital logic, AI, and analog chemical processes offers a promising route toward distributed control and on-demand transformation without reliance on centralized computation or external control units.

At the core of this evolution is the powerful intersection of chemistry, mechanics, and machine intelligence. When these domains are harmonized, they unlock capabilities that none could achieve alone materials that bend in response to ions, stretch under chemical gradients, autonomously evolve through AI-guided feedback, or reconfigure their stiffness based on predictive algorithms. Among these, mechanochemically active systems particularly mechanophores and reversible supramolecular assemblies are expected to play a central role in near-term industrial adoption due to their relatively well-established synthetic routes and compatibility with existing polymer processing technologies. As this field continues to evolve, it holds the potential to redefine what materials can do, not just as structural elements, but as active, learning, and adaptive participants in their environment.

## Supplementary information

Not Applicable.

## Ethical approval

Not Applicable.

## Fundings

No funding was received for conducting this study.

## CRediT authorship contribution statement

**Mitra Najafloo:** Conceptualization, Investigation, Visualization, Writing – original draft. **Leila Naji:** Supervision, Validation, Writing – review & editing. **Christoph Eberl:** Supervision, Validation, Writing – review & editing.

## Declaration of competing interest

The authors declare that they have no known competing financial interests or personal relationships that could have appeared to influence the work reported in this paper.

## Data Availability

Data will be made available on request.
